# Chlorine‐Free Depolymerization of Commercial PMMA via Oxygen‐Centered Radicals

**DOI:** 10.1002/anie.6957018

**Published:** 2026-07-27

**Authors:** Chih‐Yin Lin, Stelios Andreou, Nethmi De Alwis Watuthanthrige, Hyun Suk Wang, Maria‐Nefeli Antonopoulou, Athina Anastasaki

**Affiliations:** ^1^ Laboratory of Sustainable Polymers Department of Materials ETH Zurich Zürich Switzerland

**Keywords:** depolymerization, hydrogen atom transfer, photocatalyst

## Abstract

Mid‐chain depolymerization enables chemical recycling of conventional plastics with carbon–carbon backbones into high‐purity monomers under mild conditions and without any weak bonds typically installed by controlled radical polymerization. However, current reports of mid‐chain depolymerization either suffer from poor reaction yields or rely on an excess of chlorinated solvents to generate only a small fraction of chlorinated radicals with corrosive byproducts. Herein, we introduce a chlorine‐free photothermal mid‐chain depolymerization methodology, which proceeds through a distinct oxygen‐centered radical pathway. In the presence of ppm concentrations of anthraquinone, an efficient photocatalyst, up to 97% monomer can be successfully regenerated, while varying the catalyst loading allows the depolymerization rate to be modulated on demand. Notably, a wide range of commercial materials, such as Plexiglas, acrylic tubes, and light guide plates, can undergo efficient depolymerization, while temporal control could also be demonstrated via iterative “on/off” cycles.

## Graphical abstract

A new chlorine‐free photothermal method enables sustainable recycling of plastics with C–C backbones under mild conditions. Driven by an oxygen‐centered radical pathway with ppm levels of anthraquinone catalyst, it eliminates hazardous chlorinated solvents, achieving an impressive 97% depolymerization conversion. The system offers precise “on/off” light control and successfully depolymerizes commercial products like Plexiglas.

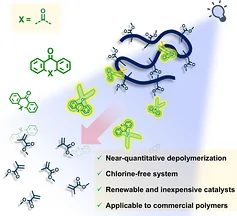

## 1 Introduction

Chemical recycling is an attractive strategy to reduce plastic waste by establishing a circular polymer economy, while overcoming product deterioration typically caused in mechanical recycling [1]. Although the high chemical stability of vinyl polymers containing carbon–carbon backbones enables them to fulfill a range of mechanical and thermal properties, the absence of weak links also hinders their depolymerization. Industrially, pyrolysis is a common approach utilized to break these bonds at high temperatures (>400°C) with undesirable byproducts and energy waste often cited as two drawbacks of the process [2, 3, 4]. To install weak links and facilitate a lower‐temperature depolymerization, reversible deactivation radical polymerization (RDRP) has recently been employed [3, 5]. Atom transfer radical polymerization (ATRP) and reversible addition‐fragmentation chain‐transfer (RAFT) polymerization have enabled the incorporation of a halogen and thiocarbonylthio group, respectively, in polymethacrylates and polymethacrylamides allowing for depolymerization to proceed at lower temperatures, typically between 120°C and 170°C [6, 7, 8, 9, 10, 11, 12, 13, 14, 15]. Apart from exclusively thermal depolymerizations, alternative external stimuli have also been explored such as electricity, light, as well as the use of radical initiators in both the ATRP and RAFT arena [16, 17, 18, 19, 20, 21, 22, 23, 24, 25, 26, 27]. However, regardless of the stimulus selected, the overwhelming majority of current reports are limited to chain‐end depolymerizations, whereby the preinstalled weak links via ATRP or RAFT polymerization lower the overall stability of these materials, thus increasing their overall depolymerizability. Furthermore, these methodologies face significant hurdles: they are limited to low molecular weight polymers, are hindered by the presence of comonomers, and remain incompatible with commercial‐grade plastics. Consequently, they cannot offer a viable solution to the global plastic waste crisis. To overcome these issues, our group recently reported the chemical recycling of commercial polymethacrylates via a photothermal, main chain‐initiated pathway [28]. The proposed mechanism suggests that depolymerization is initiated by the generation of Cl radicals from the solvent, followed by hydrogen atom transfer (HAT) from the polymer backbone and subsequent β‐scission. However, due to the low absorptivity of the chlorinated solvent, 1,2‐dichlorobenzene (DCB), in the 365–415 nm range, a large excess of solvent was required to ensure sufficient radical generation. While this approach successfully regenerated high percentages of monomer, the large excess of chlorinated solvents remains a significant drawback. Also in chlorinated solvents, Boyer and coworkers reported the depolymerization of PMMA in the presence of RAFT agents, achieving monomer recovery of up to 54% [29]. Freakley and coworkers sought to replace DCB with non‐chlorinated alternatives, however only moderate conversions could be achieved (e.g., 30%–50%) and DCB still proved the best solvent choice, enabling high depolymerization yields [30]. Benzonitrile, in particular, proved to be a promising candidate; although the initiation mechanism remains unclear and only 28% conversion was achieved, these results inspired our current study and laid the foundation of our work. Furthermore, a bulk chemical recycling approach has recently been published by the Sumerlin group, whereby, through a clever two‐step post‐modification process, up to 70% monomer can be recovered at 260°C [31].

**FIGURE 1:**
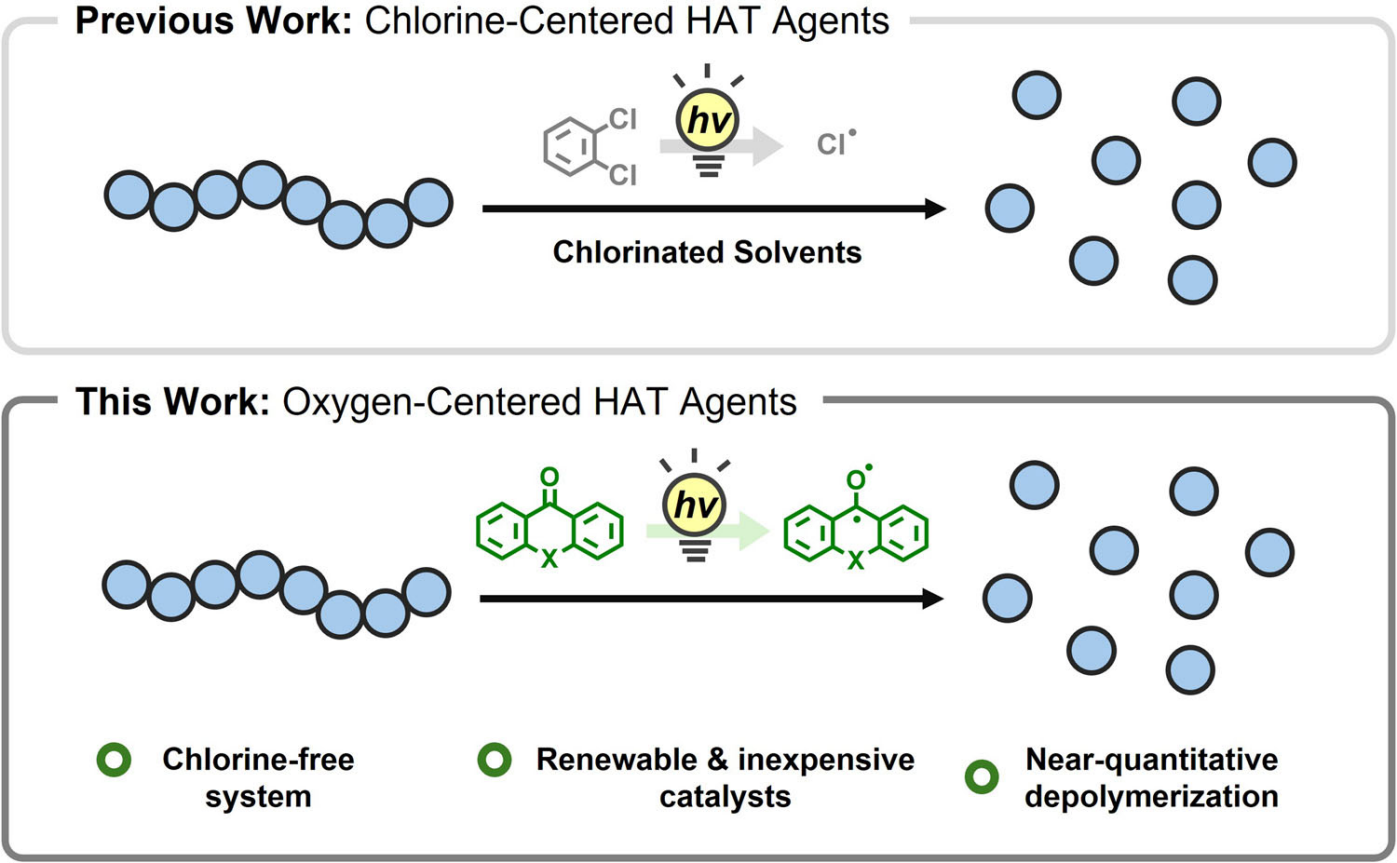
Conceptual scheme on the depolymerization of PMMA triggered by chlorine‐centered radicals (previous work) versus oxygen‐centered radicals (this approach).

Herein, we report an efficient depolymerization methodology, which proceeds in the complete absence of both chlorinated solvents and chlorine radicals without compromising reaction yields (Figure 1). A wide range of photocatalysts has been successfully screened, revealing anthraquinone as a highly efficient photocatalyst, resulting in nearly quantitative depolymerizations (>97%). Other benefits of our approach include the distinct mechanistic pathway whereby the HAT reaction is triggered by oxygen‐centered radicals, the possibility to regulate the depolymerization rate by simply tuning the photocatalyst concentration, and the excellent temporal control demonstrated by iterative “on/off” cycles.

## 2 Results and Discussion

**FIGURE 2:**
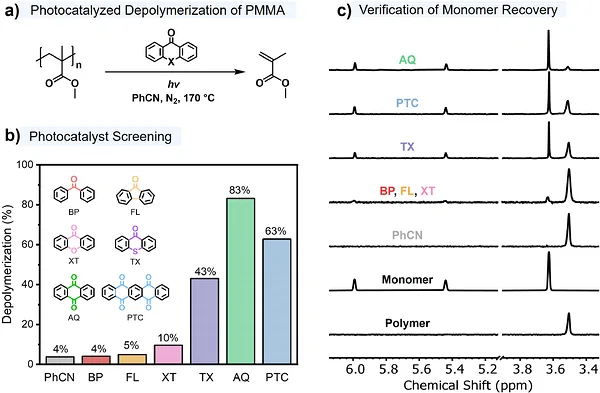
(a) Reaction scheme for PMMA depolymerization. (b) Photocatalyst screening across various aromatic ketones (RU: photocatalyst = 1:0.025) where PhCN = Benzonitrile, BP = Benzophenone, FL = 9‐Fluorenone, TX = Thioxanthone, XT = Xanthone, AQ = Anthraquinone, and PTC = 5,7,12,14‐Pentacenetetrone. (c) Verification of monomer regeneration via ^1^H NMR spectroscopy.

Prior to initiating our depolymerization studies, polymethylmethacrylate (PMMA), was synthesized as a model material via conventional radical polymerization (*M*_n_ = 320,000, *Đ* = 1.8, Figure S1). We envisioned that the development of a photothermal depolymerization pathway in the presence of an efficient direct HAT photocatalyst would potentially eliminate the use of chlorinated solvents, as well as other chlorinated compounds, including chlorinated catalysts, without sacrificing the reaction yield [32]. Initially, we sought to identify a suitable solvent that would fulfill a number of criteria (Figure S2). First, the solvent must be compatible with HAT chemistry (i.e., contain strong C─H bonds), in order to host a successful depolymerization, while minimizing solvent‐induced side reactions. Second, a solvent with a relatively high boiling point would be advantageous to avoid high‐pressure reaction vessels at moderate reaction temperatures. Third, the ideal solvent must offer sufficient stabilization of triplet‐state radicals to prolong their lifetimes, thereby maximizing their reactivity toward the polymer backbone [33, 34]. In search of such requirements, and inspired by a previous report by Husband et al., benzonitrile was explored as an exemplary polar aprotic solvent [30]. To start our investigation, a control experiment was performed whereby PMMA dissolved in benzonitrile was irradiated at 365 nm and heated at 170°C in the absence of a photocatalyst (10 mM repeat unit concentration, Figure 2). No meaningful depolymerization was detected by either nuclear magnetic resonance (^1^H NMR) or size exclusion chromatography (SEC), even when the reaction was left to proceed for 24 h (Figures 2b and S3). This is in contrast to a previously published report by Husband et al. in which a 28% conversion in benzonitrile was observed and attributed to the formation of CN and/or aryl radicals [30]. We realized that the key difference is that we purified the solvent prior to use by passing it through a column of basic alumina. Indeed, when unpurified benzonitrile was instead employed, 30% conversion was achieved, which is on par with the value reported by Husband et al. (Figure S4). As such, the conversion obtained in the absence of a photocatalyst is exclusively attributed to solvent impurities identified through gas chromatography coupled with mass spectrometry (GC‐MS) analysis (Figures S5 and S6), and we continued our study utilizing purified benzonitrile. Inspired by previous work on photocatalyzed polymerizations, and HAT processes in small‐molecule systems, benzophenone (BP) was initially selected for its widespread use (0.025 equiv. with respect to repeat unit concentration) [35, 36, 37, 38].

However, no meaningful depolymerization could be detected by either NMR or SEC analysis, even when the reaction was left to proceed overnight irradiation at 365 nm (Figure 2b). This can be attributed to BP's structural flexibility, where unrestricted phenyl ring rotation at 170°C shortens the reactive triplet‐state lifetime via non‐radiative decay, limiting productive HAT [39, 40, 41]. To address this, we then switched to a more planar and rigid analogue, fluorenone (FL), intended to stabilize the excited state and prolong the radical lifetime [42], which resulted in a small percentage of regenerated monomer (5%). Xanthone (XT) and thioxanthone (TX) were subsequently screened to examine how heteroatom inclusion modulates the electronic density of the aromatic scaffold. Consistent with our design strategy, the transition to these catalysts with higher triplet energies and improved absorbance at 365 nm (Table S2 and Figure S7) increased monomer recovery to 10% and 43%, validating the targeted optimization of the photocatalyst structure. It was hypothesized that an additional carbonyl group could enhance the photocatalytic activity by extending the π‐conjugation and improving radical stabilization [43]. To investigate this, anthraquinone (AQ) was employed. Pleasingly, and in line with our hypothesis, a striking 83% monomer was successfully regenerated, highlighting AQ as an efficient depolymerization photocatalyst. Notably, despite its lower absorbance at 365 nm, AQ displayed high photocatalytic activity, consistent with findings previously reported by Barner–Kowollik and coworkers, demonstrating a fundamental mismatch between absorptivity and photochemical reactivity [44]. However, when the conjugation was further extended in 5,7,12,14‐pentacenetetrone (PTC), a lower conversion (i.e., 63%) was detected. It is noted that for all cases, we not only confirmed depolymerization through SEC (Figure S8 and Table S3), but also directly observed monomer regeneration by NMR (Figure 2c).

**FIGURE 3:**
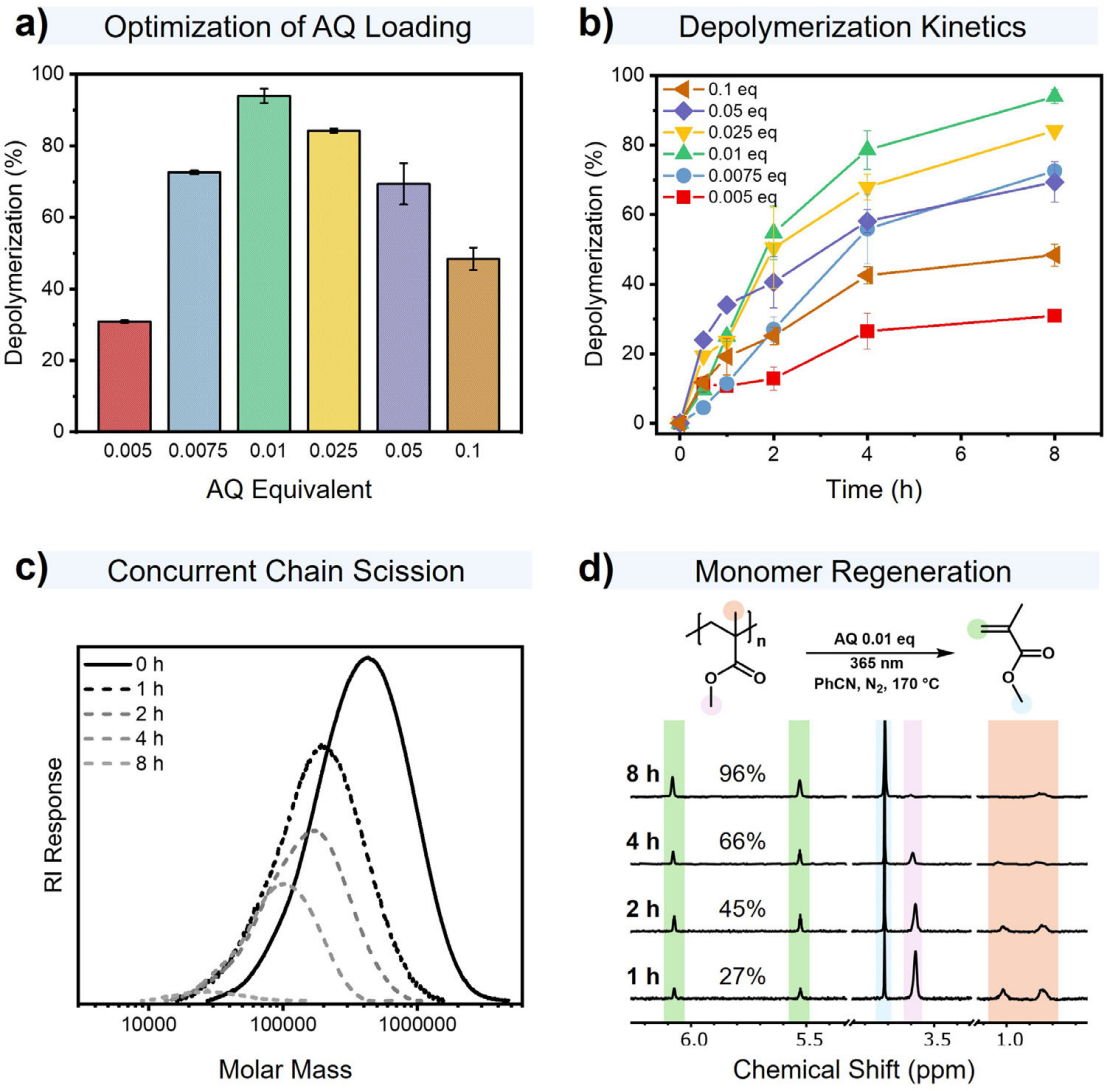
(a) Effect of anthraquinone loading on depolymerization (RU:AQ = 1:x, where *x* = 0.005–0.1; 170°C in PhCN); (b) Depolymerization kinetics as a function of photocatalyst concentration; (c) SEC traces illustrating concurrent chain scission and depolymerization; (d) Monomer regeneration kinetics as confirmed by ^1^H NMR spectroscopy.

Considering that AQ showed the most encouraging data, we subsequently sought to optimize the photocatalyst loading (Figure 3a and Table S4). Upon increasing the photocatalyst concentration from 0.025 equiv. to 0.05 and 0.1 equiv., the conversion dropped to 69% and 48%, respectively, although significant shifts in the SEC traces were still observed (Figure S9). To investigate the diminished conversion observed at higher catalyst loadings, we evaluated catalyst solubility, light attenuation, and potential side reactions. Across all tested concentrations, AQ remained fully soluble, ruling out macroscopic solution turbidity or light scattering as contributing factors (Figure S10a). In contrast, UV–vis analysis revealed a 58% transmittance at 0.1 equiv., confirming partial light attenuation via the inner filter effect (Figure S10b,c). Furthermore, distinct UV signals within the SEC traces of the residual polymer confirmed the occurrence of side reactions involving polymer radicals with the increased AQ loadings (Figure S11). Consequently, we hypothesize that this decreased efficiency stems from a synergy between partial light attenuation and competitive radical termination events.

With this in mind, the catalyst was decreased to 0.01 equiv., resulting in a conversion of 96% (Figure S12). It is noted that this is the highest conversion ever reported in a non‐chlorinated solvent and is on par with the best conversions previously achieved in chlorinated solvents [28]. At even lower catalyst loadings, the conversion decreased, indicating that radical generation was insufficient to sustain the HAT reaction. Nevertheless, by varying the photocatalyst concentration, the depolymerization rate could be fully regulated (Figure 3b), whereas in previous work, the reaction rate was strictly dependent on the rate of solvent radical formation [28, 30]. SEC was also employed to monitor the reaction (Figure 3c and Table S5), revealing chain scission occurring concurrently with an efficient depolymerization, resulting in the formation of pure monomer (Figure 3d).

**FIGURE 4:**
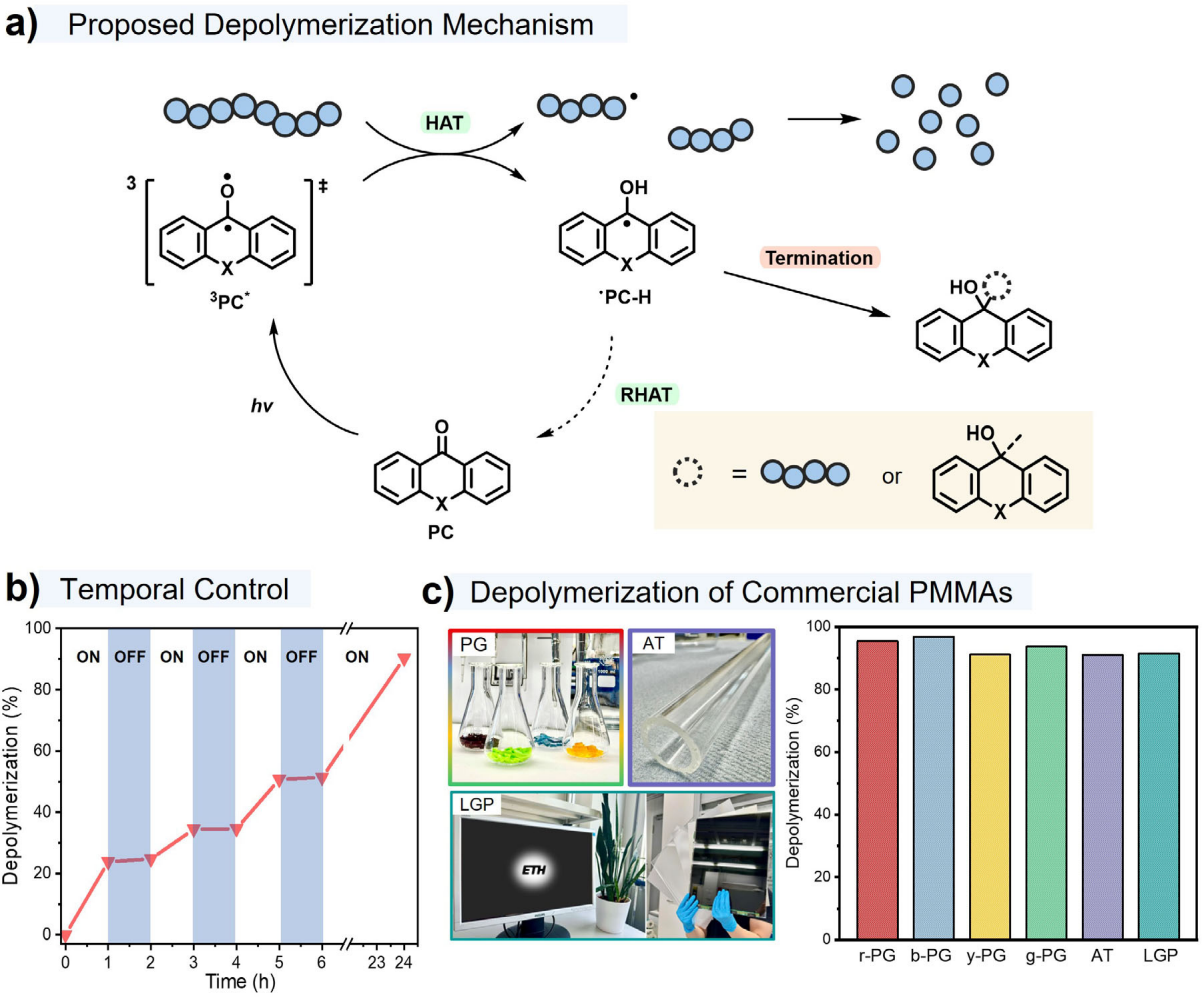
(a) Proposed HAT‐mediated mechanism for PMMA depolymerization using aromatic ketones; (b) Temporal control of depolymerization: Kinetic plots showing reaction progress during stimulus “on” and “off” cycles; (c) Left: photographs of colored Plexiglas (top left‐ r‐PG: red, b‐PG: blue, y‐PG: yellow, g‐PG: green), acrylic tubes (AT, top right), and LCD light guide plates (LGP, bottom), and right: depolymerization conversions across different commercial PMMA grades.

Taken altogether, a proposed mechanism for our depolymerization pathway is illustrated in Figure 4a (Figure S13). In line with the literature, whereby aromatic ketones have been routinely employed as HAT agents [45, 46, 47], it is postulated that upon irradiation, the photocatalyst (PC) is excited to its singlet state, followed by efficient intersystem crossing (ISC) to yield a triplet biradical state (^3^PC^*^) [34, 48, 49, 50, 51, 52, 53]. This species, possessing highly electrophilic oxygen‐radical character (Table S6), abstracts a hydrogen atom from either the methyl or methylene units of the PMMA backbone (Table S7). The resulting carbon‐centered macroradical subsequently undergoes β‐scission, followed by depolymerization under thermodynamically favorable conditions. Following the HAT process, a carbon‐centered ketyl radical forms on the photocatalyst (^•^PC‐H); this intermediate can either undergo dimerization with another radical species (^•^PC‐H) or termination with a polymer radical [36, 49].

While the existing literature reports that AQ regeneration is possible in the presence of oxygen [48], our N_2_ degassing protocol reduces O_2_ levels in the reaction mixture to below detectable limits (Figure S14). We therefore hypothesized that PC regeneration may happen through a reverse HAT (RHAT) process, potentially facilitated by the presence of hydrogen acceptors (polymer radicals) or via intermolecular disproportionation with another PC molecule (Figure S15), or the presence of a trace amount of residual oxygen [43, 54, 55]. As such, our developed methodology not only avoids the use of chlorinated solvents and compounds but also proceeds via a distinct mechanism whereby HAT is performed through oxygen‐centered radicals, rather than Cl radicals.

Next, we investigated the possibility of temporal control during the depolymerization via intermittent “on/off” cycles (Figure 4b). Upon switching the light “on” the depolymerization was initiated, reaching 24% of conversion within 1 h. A nearly complete discontinuation of the depolymerization was then observed when the light source was removed. On re‐exposing the reaction vessel under light irradiation at 170°C, the original depolymerization rate was restored. These cycles were repeated several times without affecting either the reaction rate or the final depolymerization conversion, thus highlighting the robustness of this methodology.

Finally, we were interested in exploring the applicability of our system to commercial Plexiglas, typically containing a range of impurities, including dyes, additives, and comonomers. For this purpose, we purchased various Plexiglas materials consisting of different colors and transparency, as well as acrylic tubes (AT) and light guide plates (LGP), as can be seen in Figure 4c. Without any prior purification, and under previously judiciously optimized reaction conditions, nearly quantitative depolymerization was observed (up to 97%, Table S8). Notably, this high efficiency was maintained upon scale‐up, enabling the isolation of the regenerated monomer in high purity (Figures S16 and S17). Together, these findings validate the versatility of our approach.

## 3 Conclusion

To summarize, a completely chlorine‐free depolymerization of PMMA synthesized by conventional radical polymerization was developed. Unlike previous methodologies that rely on chlorine radicals, the presence of a highly efficient photocatalyst, namely AQ, enabled the formation of oxygen‐centered radicals, which then acted as HAT agents to abstract a hydrogen from the polymer backbone, resulting in concurrent scission and depolymerization up to 97%. The method could also be directly applicable to commercial materials, while excellent temporal control was demonstrated through intermittent “on/off” cycles.

## Author Contributions

**Chih‐Yin Lin**: writing – original draft, visualization, methodology, formal analysis, investigation, conceptualization. **Stelios Andreou**: investigation, writing – review and editing. **Nethmi De Alwis Watuthanthrige**: conceptualization, writing – review and editing, supervision, investigation. **Hyun Suk Wang**: writing – review and editing, supervision. **Maria** – **Nefeli Antonopoulou**: supervision, writing – review and editing. **Athina Anastasaki**: conceptualization, writing – review and editing, supervision, funding acquisition, project administration.

## Conflicts of Interest

The authors declare no conflicts of interest.

## Supporting information



The data supporting the findings of this study are available in the paper and its Supporting Information. The authors have cited additional references within the Supporting Information [52, 56, 57].
**Supporting File**: anie73796‐sup‐0001‐SuppMat.pdf.

## Data Availability

The data that supports the findings of this study are available in the Supporting Information of this article.
