# Supplementary material for: Chlorine‐Free Depolymerization of Commercial PMMA via Oxygen‐Centered Radicals
Source: Angew Chem Int Ed Engl. 2026 Jul 27;65(38):e6957018. doi: 10.1002/anie.6957018 (PMC13573122; doi:10.1002/anie.6957018)
Supplement: Supplementary file 1 [file ANIE-65-e6957018-s001.pdf]

# Chlorine-Free Depolymerization of Commercial PMMA via Oxygen-Centered Radicals

Chih-Yin Lin,[a] Stelios Andreou,[a] Nethmi De Alwis Watuthanthrige,[a] Hyun Suk Wang,[a] Maria-Nefeli Antonopoulou,[a] and Athina Anastasaki*[a]

[a] Chih-Yin Lin, Stelios Andreou, Nethmi De Alwis Watuthanthrige, Hyun Suk Wang, Maria-Nefeli Antonopoulou, and Athina Anastasaki,
Laboratory of Sustainable Polymers, Department of Materials,
ETH Zurich, Zürich 8093, Switzerland
athina.anastasaki@mat.ethz.ch

## Table of Contents

**1 Materials and Instrumentation** .......... **5**

**1.1 Materials** .......... 5

**1.2 Instruments** .......... 5

**1.2.1 Size Exclusion Chromatography (SEC)** .......... 5

**1.2.2 $^{1}$H NMR** .......... 6

**1.2.3 Ultraviolet-Visible (UV-Vis) Spectroscopy** .......... 6

**1.2.4 Gas-Chromatography coupled with Mass Spectrometry (GC-MS)** .......... 6

**1.2.5 Measurements of $O_2$ Concentrations** .......... 6

**1.2.6 Setup of Depolymerization Reactor** .......... 7

**1.2.7 AQ Solution Transmittance Calculation** .......... 7

**2 Experimental** .......... **8**

**2.1 Synthesis Procedures** .......... 8

**2.1.1 Free Radical Polymerization (FRP) of PMMA** .......... 8

**2.2 Depolymerization Procedures** .......... 8

**2.2.1 Depolymerization of PMMA Synthesized via FRP for Photocatalyst Screening** .......... 8

2.2.1.1 Depolymerization of PMMA with Benzophenone (BP) .......... 8

2.2.1.2 Depolymerization of PMMA with 9-Fluorenone (FL) .......... 9

2.2.1.3 Depolymerization of PMMA with Xanthone (XT) .......... 9

2.2.1.4 Depolymerization of PMMA with Thioxanthone (TX) .......... 9

2.2.1.5 Depolymerization of PMMA with Anthraquinone (AQ) .......... 9

2.2.1.6 Depolymerization of PMMA with 5,7,12,14-Pentacenetetrone (PTC) .......... 10

2.2.1.7 Control Experiment: Depolymerization of PMMA without Photocatalyst .......... 10

**2.2.2 Optimization of AQ Loading in Depolymerization of FRP-PMMA** .......... 10

2.2.2.1 Initial Screening .......... 10

2.2.2.2 Kinetic Experiments .......... 11

**2.2.3 Depolymerization of the Commercial PMMAs** .......... 12

2.2.3.1 Monomer Isolation .......... 12

**2.2.4 Temporal Control of Depolymerization of PMMA** .......... 14

**3 Additional Data** .......... **14**

**3.1 Characterization of PMMA Synthesized via FRP** .......... 14

**3.2 Solvent Selection for HAT Reaction** .......... 15

**3.3 Control Experiments – Depolymerization without AQ** .......... 15

**3.4 Photochemical Properties of Aromatic Ketones** .......... 18

3.5 **Photocatalyst Screening for Depolymerization of PMMA** .......... 18
3.6 **Optimization of AQ Loadings** .......... 19
3.7 **Kinetic Profile of the Depolymerization with Optimized AQ Loading** .......... 22
3.8 **Proposed Mechanism of Oxygen-Centered HAT Agent Triggered Mid-Chain Depolymerization of PMMA** .......... 24
3.9 **Radical Electrophilicity and Bond Dissociation Energies** .......... 25
3.10 **Depolymerization of Commercial PMMAs** .......... 26
**4 References .......... 28**

## List of Figures

**Figure S1** Characterization of PMMA: a) SEC trace ($M_n$ = 320,000, Đ = 1.8), b) $^1$H NMR spectrum after purification. .......... 14
**Figure S2** Venn diagram for solvent selection .......... 15
**Figure S3** Control experiment with purified benzonitrile. RUC of 10 mM PMMA in benzonitrile was reacted for 23 h at 170 °C with 365 nm light. a) SEC traces of $t_{0h}$ and $t_{23h}$. b) $^1$H NMR showed a negligible amount of monomer recovery. .......... 15
**Figure S4** Control experiment with unpurified benzonitrile (unpurified PhCN) and purified benzonitrile (purified PhCN). RUC of 10 mM PMMA in benzonitrile was reacted for 23 h at 170 °C with 365 nm light. a) Depolymerization conversion measured by SEC. b) $^1$H NMR showed approximately 30% monomer regeneration with unpurified PhCN, while nearly no monomer was recovered with purified PhCN. .......... 16
**Figure S5** Evidence for removal of impurities in benzonitrile. a) Photographs of residues remaining after air-drying 100 mL of benzonitrile without purification (left) and after purification with basic alumina (right). b) Gas chromatogram of unpurified benzonitrile, showing three main identified compounds. c) Gas chromatogram of purified benzonitrile. .......... 16
**Figure S6** Identification of impurities in benzonitrile. a) Mass spectrum corresponding to the first peak in the unpurified benzonitrile gas chromatogram. b) Mass spectrum corresponding to the second peak in the unpurified benzonitrile gas chromatogram. c) Mass spectrum corresponds to the third peak in the unpurified benzonitrile gas chromatogram. .......... 17
**Figure S7** UV-vis spectra of the aromatic ketones and benzonitrile. Baseline for aromatic ketones was benzonitrile, and baseline for benzonitrile was air. Solution concentrations were 0.3 mM for BP, FL, TX, AQ, and PTC, and 0.17 mM for XT. .......... 18
**Figure S8** Evolution of molecular weight distributions during the aromatic ketone-mediated depolymerization of PMMA, as monitored by SEC. RUC of 10 mM PMMA and 0.025 eq aromatic ketones in benzonitrile were reacted for 8 h at 170 °C with 365 nm light. Significant concurrent chain scission and depolymerization happened in the reaction with TX, AQ, and PTC. .......... 19

**Figure S9** SEC curves of depolymerization with varying amounts of AQ. RUC of 10 mM PMMA and 0.005-0.1 eq aromatic ketones in benzonitrile were reacted for 8 h at 170 °C with 365 nm light. ........ 20
**Figure S10** Solubility and transmittance profiles of AQ in benzonitrile. (a) Visual appearance of the reaction mixtures showing full catalyst solubility across all tested equivalents. (b) UV-Vis absorption spectrum of AQ in benzonitrile; the target excitation wavelength (365 nm) is highlighted in blue. (c) Plot illustrating the relationship between depolymerization efficiency and solution transmittance at 365 nm as a function of catalyst loading. ........ 21
**Figure S11** SEC traces of the polymers after 24 h of reaction across catalyst loadings from 0.01 to 0.1 equiv of AQ. The increase in the UV signal intensity relative to the refractive index (RI) traces indicates the incorporation of UV-active molecules into the polymer backbone. ........ 22
**Figure S12** Depolymerization profile of RUC PMMA. Conditions: 10 mM RUC PMMA in benzonitrile at 170 °C, 0.01 eq. AQ, under 365 nm irradiation. (a) Kinetic profile comparing monomer conversions determined via SEC and $^{1}$H NMR spectroscopy (inset: $^{1}$H NMR spectra of the reaction mixture before (0 h) and after (8 h) depolymerization). (b) $^{1}$H NMR spectra showing the evolution of the polymer methoxy peaks (3.6–3.8 ppm, integrated to 300) and the emerging vinyl peaks of the regenerated MMA monomer (5.5 and 6.1 ppm) at 1, 2, 4, and 8 h, corresponding to 27%, 45%, 67%, and 96% conversion, respectively. ........ 23
**Figure S13** Proposed mechanism for mid-chain scission and depolymerization via triplet biradical with oxygen-centered reactivity derived from aromatic ketones. ........ 24
**Figure S14** Dissolved oxygen concentration as a function of $N_2$ purging time during the degassing phase prior to reaction (15min). ........ 25
**Figure S15**: Proposed AQ regeneration pathways. a) Reverse HAT process with a polymeric radical, b) Disproportionation of the ·PC-H with another PC molecule ........ 25
**Figure S16** Isolation of monomer. (a) *In situ* distillation apparatus used for the recovery of monomer during depolymerization under 365 nm light at 170 °C (reaction conditions: 750 mM [RUC ], 0.01 eq. AQ). (b) Photograph of the collected liquid monomer (1.08 g) in a vial. ........ 27
**Figure S17** Characterization of the isolated monomer**.** Top: $^{1}$H NMR spectrum of the reaction mixture (taken under $N_2$) at 8 h after initiating the reaction. Bottom: $^{1}$H NMR spectrum of the final collected liquid in the receiving flask, confirming the isolation of MMA monomer in ~95% purity. ........ 28

**List of Tables**

**Table S1** Reaction solution preparation for optimization of AQ loading ........ 11
**Table S2** Maximum absorbance, molar absorptivity, and reported triplet energy of aromatic ketones. ........ 18
**Table S3** Depolymerization Conversions and $M_n$ (at $t_{0h}$ and $t_{8h}$) of Photocatalyst Screening§ ........ 18
**Table S4** Evolution of depolymerization conversion and $M_n$ compared to the initial polymer for the depolymerization of PMMA using X (where X = 0.005-0.1) eq AQ § ........ 20

**Table S5** Depolymerization conversions and molecular weight evolution for the depolymerization of PMMA using 0.01 eq AQ.§ ........ 22
**Table S6** Electrophilicity of chlorine radical and triplet biradical with oxygen-centered reactivity.[2] ........ 25
**Table S7** Comparison of C–H bond dissociation energies (BDE) across diverse chemical environments. [3] ........ 26
**Table S8** Depolymerization Conversion and $M_n$ of Depolymerization of Commercial PMMAs 26

# 1 Materials and Instrumentation

## 1.1 Materials

All materials were obtained from Sigma Aldrich, Fischer Scientific, or TCI and used as received, while those were pretreated or from other sources were stated below separately. Benzonitrile and methyl methacrylate were purified via passing through basic alumina column before using. Benzonitrile was purchased from Fischer Scientific. Anthraquinone was from Combiblocks (Combi-Blocks, Inc. (CBL)). 365 nm LED strips (real UV™) were purchased from Waveform Lighting. Poly(methyl methacrylate) (PMMA) standards for calibration of SEC were purchased from Aglient Technologies (PMMA EasiVials). For the commercial PMMAs, PLEXIGLAS® sheets were purchased from POLYVANTIS, acrylic tubes were from Werkstarck, and LGP was from the LCD monitor from Acer.

## 1.2 Instruments

### 1.2.1 Size Exclusion Chromatography (SEC)

Size exclusion chromatography was performed on a Shimadzu instrument, equipping with a SIL-20A HT automatic sampler, LC-20AD pump liquid chromatogram, CBM-20A communication module, and CTO-20A column oven to maintain SEC measurement temperature at 40 °C (Columns: 50 x 7.5 mm guard column with 10 µm bead size, and three 300 x 8 mm KF-805L columns with 10 µm bead size, which pore size maximum is 5000 Å). SPD-20A detector and RID-20A detector were used for UV/VIS and refractive index recording respectively. N, N-dimethylacetamide (DMAc, Acros, HPLC grade, with 0.03 w/v LiBr) was used as eluent and the flow rate was set to 1 mL/min. Poly(methyl methacrylate) standards were used for calibration with the molecular weight range from 7,000 to 1,600,000 g/mol. All SEC samples were dissolved in DMAc and filtered by 0.45 µm PTFE syringe prior to analysis.

### 1.2.2 $^{1}H$ NMR

A Bruker Avance-400 MHz spectrometer with a cryo-probe was used for NMR analysis. $CDCl_3$ or acetone-$d_6$ were used as NMR solvents. Chemical shifts are shown in ppm downfield from tetramethylsilane (TMS) and referenced to residual solvent proton signals.

### 1.2.3 Ultraviolet-Visible (UV-Vis) Spectroscopy

V-730 UV-visible spectrophotometer was used to obtain the UV-vis absorption data at room temperature. The aromatic ketones were dissolved in benzonitrile and transferred to a standard 10 mm path-length quartz cuvette. Benzonitrile was used as the baseline for all measurements.

### 1.2.4 Gas-Chromatography coupled with Mass Spectrometry (GC-MS)

Thermo Scientific QExative GC Orbitrap with Ion-Trap (Orbitrap) and electron impact (EI) as ion source was used. Mass spectrometry experiments were operated on a Bruker Maxis I quadrupole-time-of-flight (QTOF) at 4500V emitter voltage with electrospray ionization (ESI) as ion source.

To prepare the samples, 100 mL benzonitrile before or after purification were dried with air blowing in a 20 mL vial to isolate the solid impurities. The solid residue was then transferred to the sampling tube for GC-MS analysis.

### 1.2.5 Measurements of $O_2$ Concentrations

A Pocket Oxygen Meter - FireStingGO2 (from Pyro Science) was used for all measurements. The solvent-resistant oxygen probe OXSOLV measures oxygen partial pressure in various polar and nonpolar solvents, based on optical detection principles (REDFLASH technology). The fiber-optic oxygen sensor tip is covered with a stainless-steel tube 1.5 mm in diameter and 150 (or 40) mm in length. The analysis of the data was conducted with the FireStingGO2 Manager software.

To monitor oxygen levels, we inserted an oxygen probe through a rubber septum into the reaction vessel, ensuring a sealed system. Continuous $O_2$ concentration measurements were initiated at the start of $N_2$ degassing and recorded at 1-minute intervals over a 15-minute period, which represents the standard degassing time prior to a single experiment.

### 1.2.6 Setup of Depolymerization Reactor

A 100-inch LED strip was attached to a cork ring (Diameter = 9 cm) and placed to surround a 250 mL glass beaker (Diameter = 6 cm) (Intensity of the light = 23 mw/cm$^2$). The reaction tube was placed in the oil bath in the beaker with the distance of approximately 1.5 cm to each LED as illustrated in **Scheme S 1**. Photoreactor intensity was determined using a Thorlabs PM16-401D light power meter with integrating sphere photodiode sensor (Wavelength range = 350 - 1100 nm, Optical Power Range = 1 μW - 500 mW).

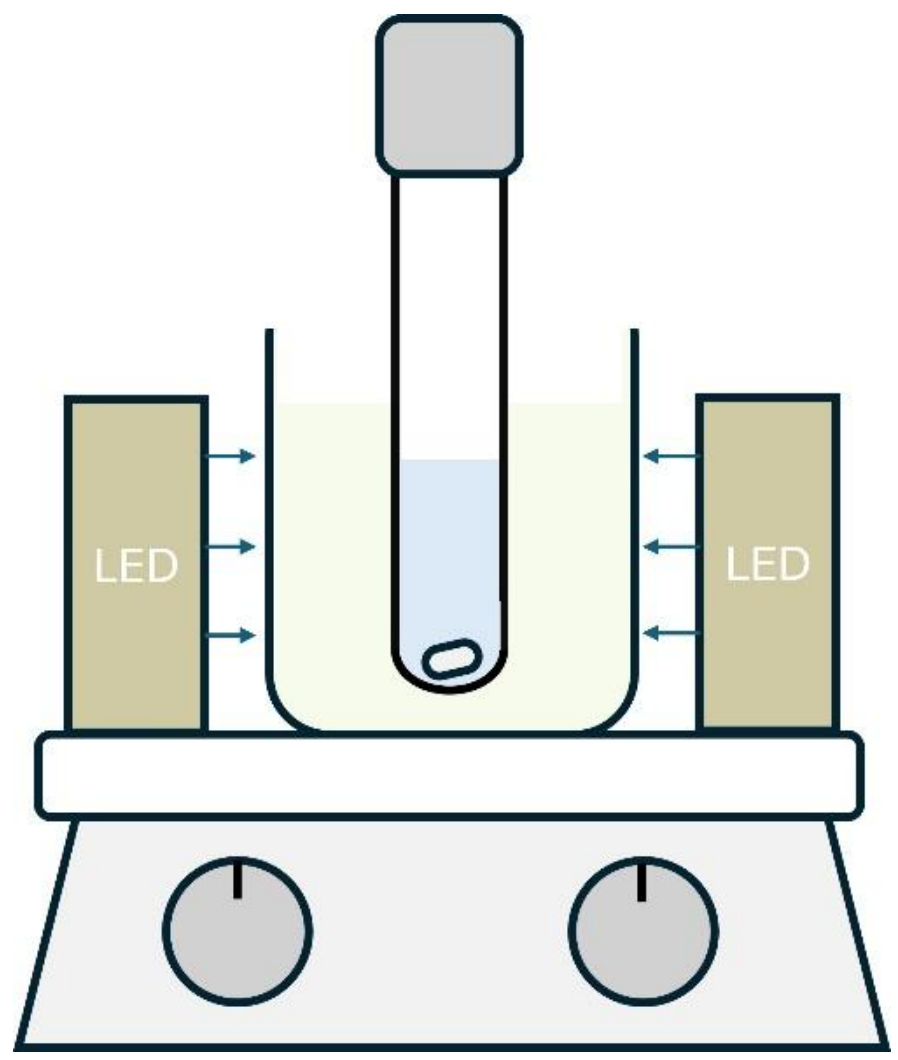


**Scheme S1** Depolymerization Reactor Setup

### 1.2.7 AQ Solution Transmittance Calculation

0.05 to 1 mM AQ solution were prepared for the UV-vis measurement, as 0.1 mM solution was described below as representative. 2.1 mg of AQ was dissolved in 5 mL of benzonitrile. 0.15 mL AQ stock solution and 2.85 mL benzonitrile were mixed in a 5 mL glass vial and then transferred to the quartz cuvette for UV-vis analysis.

The absorbance measured at 365 nm by UV-vis was utilized to determine the transmittance of the AQ solution of each concentration, using the equation shown below.

$$\boldsymbol{T}(\%) = \mathbf{10^{2-A}}$$ ,where T is transmittance, A is absorbance.

# 2 Experimental

## 2.1 Synthesis Procedures

### 2.1.1 Free Radical Polymerization (FRP) of PMMA

A volume of 6 mL methyl methacrylate (MMA, 5.6 g, 56.3 mmol) and 1.9 mg 2,2'-azobis(isobutyronitrile) (AIBN, 11.7 μmol) were added into 12 mL acetonitrile in a 50 mL round-bottom flask; a stir bar was put in the flask before being sealed with a septum. The solution was deoxygenated by nitrogen purging for 15 minutes, and the polymerization was conducted in an oil bath at 70 °C for 15 hours (stirring rate = 400 rpm). The flask was removed from the oil bath and cooled to room temperature to stop the polymerization. The purification was done by precipitating in methanol three times, and the polymer (FRP-PMMA) was dried in a vacuum overnight and checked by $^1$H NMR to ensure there was no monomer residue.

## 2.2 Depolymerization Procedures

### 2.2.1 Depolymerization of PMMA Synthesized via FRP for Photocatalyst Screening

#### 2.2.1.1 Depolymerization of PMMA with Benzophenone (BP)

Stock solutions were prepared as described below. 20 mg of PMMA was dissolved in 10 mL benzonitrile, and 1.8 mg of BP was dissolved in 5 mL benzonitrile.

1 mL PMMA stock solution, 0.25 mL BP stock solution, and 0.75 mL benzonitrile were mixed ($[RU_0]$ = 10 mM, $RU_0$ : BP = 1 : 0.025) in a 10 mL glass tube equipped with a magnetic stir bar. The tube was sealed with a rubber septum and deoxygenated via nitrogen purging for 15 minutes. The vessel was then submerged in an oil bath preheated to 170 °C (stirring at 400 rpm), ensuring the internal liquid level remained approximately 1 cm below the oil surface. After 5 minutes of thermal equilibration, the LED strips were activated to irradiate the HAT agent. The reaction was quenched after 8 h by removing the vessel from the heat and light source and cooling to room temperature.

A 0.35 mL aliquot was taken for $^1$H NMR and SEC analyses. To prepare the SEC samples, 0.3 mL solution was transferred to a 5 mL vial, dried under a stream of air, and redissolved in 0.35 mL DMAc. The resulting solution was filtered through a 0.45 μm PTFE membrane

syringe filter into a vial equipped with an insert. For $^1$H NMR analysis, the remaining 50 μL aliquot was diluted in $CDCl_3$.

**2.2.1.2 Depolymerization of PMMA with 9-Fluorenone (FL)**

The depolymerization of PMMA using FL as the HAT agent was conducted following the procedure established for BP (section 2.2.1.1) with stock solutions prepared as follows:

20 mg of PMMA was dissolved in 10 mL of benzonitrile, and 1.8 mg of FL was dissolved in 5 mL of benzonitrile. 1 mL PMMA stock solution, 0.25 mL FL stock solution, and 0.75 mL benzonitrile were mixed in a 10 mL glass tube to reach the initial concentrations ($[RU_0]$ = 10 mM, $RU_0$ : FL = 1 : 0.025).

**2.2.1.3 Depolymerization of PMMA with Xanthone (XT)**

The depolymerization of PMMA using XT as the HAT agent was conducted following the procedure established for BP (section 2.2.1.1) with stock solutions prepared as follows:

20 mg of PMMA was dissolved in 10 mL of benzonitrile, and 2.0 mg of XT was dissolved in 5 mL of benzonitrile. 1 mL PMMA stock solution, 0.25 mL XT stock solution, and 0.75 mL benzonitrile were mixed in a 10 mL glass tube to reach the initial concentrations ($[RU_0]$ = 10 mM, $RU_0$ : XT = 1 : 0.025).

**2.2.1.4 Depolymerization of PMMA with Thioxanthone (TX)**

The depolymerization of PMMA using TX as the HAT agent was conducted following the procedure established for BP (section 2.2.1.1) with stock solutions prepared as follows:

20 mg of PMMA was dissolved in 10 mL of benzonitrile, and 2.1 mg of TX was dissolved in 5 mL of benzonitrile. 1 mL PMMA stock solution, 0.25 mL TX stock solution, and 0.75 mL benzonitrile were mixed in a 10 mL glass tube to reach the initial concentrations ($[RU_0]$ = 10 mM, $RU_0$ : TX = 1 : 0.025).

**2.2.1.5 Depolymerization of PMMA with Anthraquinone (AQ)**

The depolymerization of PMMA using AQ as the HAT agent was conducted following the procedure established for BP (section 2.2.1.1) with stock solutions prepared as follows:

20 mg of PMMA was dissolved in 10 mL of benzonitrile, and 2.1 mg of AQ was dissolved in 5 mL of benzonitrile. 1 mL PMMA stock solution, 0.25 mL AQ stock solution, and 0.75

mL benzonitrile were mixed in a 10 mL glass tube to reach the initial concentrations ([$RU_0$] = 10 mM, $RU_0$ : AQ = 1 : 0.025).

#### 2.2.1.6 Depolymerization of PMMA with 5,7,12,14-Pentacenetetrone (PTC)

The depolymerization of PMMA using PTC as the HAT agent was conducted following the procedure established for BP (section 2.2.1.1) with stock solutions prepared as follows:

20 mg of PMMA was dissolved in 10 mL of benzonitrile, and 3.4 mg of PTC was dissolved in 5 mL of benzonitrile. 1 mL PMMA stock solution, 0.25 mL PTC stock solution, and 0.75 mL benzonitrile were mixed in a 10 mL glass tube to reach the initial concentrations ([$RU_0$] = 10 mM, $RU_0$ : PTC = 1 : 0.025).

#### 2.2.1.7 Control Experiment: Depolymerization of PMMA without Photocatalyst

The control experiment of depolymerization of PMMA was conducted following the procedure established for BP (section 2.2.1.1) with stock solutions prepared as follows:

20 mg of PMMA was dissolved in 10 mL of benzonitrile as the reactant stock. 1 mL PMMA stock solution and 1 mL benzonitrile were mixed in a 10 mL glass tube to reach the initial concentration ([$RU_0$] = 10 mM).

### 2.2.2 Optimization of AQ Loading in Depolymerization of FRP-PMMA

#### 2.2.2.1 Initial Screening

Initial screening for the optimal AQ loading in the depolymerization of PMMA was conducted following the procedure established for BP (Section 2.2.1.1), with stock solutions prepared as follows:

Reaction mixtures with varying molar equivalents were prepared by mixing these stocks as described in **Table S1**. Reaction solutions with varying loadings of AQ were prepared, 0.005 eq AQ is used as representative example. 20 mg of PMMA was dissolved in 10 mL of benzonitrile, and 2.1 mg of AQ was dissolved in 5 mL of benzonitrile. 1 mL PMMA stock solution, 0.05 mL AQ stock solution, and 0.95 mL benzonitrile were mixed in a 10 mL glass tube to reach the initial concentrations ([$RU_0$] = 10 mM, $RU_0$ : AQ = 1 : 0.005). The preparation of reaction solutions with other AQ loadings is shown in **Table S1**. The mixtures

were formulated to achieve a target initial concentration of $[RU_0]$ = 10 mM at a specific molar ratio $RU_0$ : AQ = 1 : x.

**Table S1** Reaction solution preparation for optimization of AQ loading

| AQ loading (eq) | PMMA Stock Solution (mL) | AQ Stock Solution (mL) | Benzonitrile (mL) |
|---|---|---|---|
| 0.005 | 1 | 0.05 | 0.95 |
| 0.0075 | 1 | 0.075 | 0.925 |
| 0.01 | 1 | 0.1 | 0.9 |
| 0.025 | 1 | 0.25 | 0.75 |
| 0.05 | 1 | 0.5 | 0.5 |
| 0.1 | 1 | 1 | 0 |

#### 2.2.2.2 Kinetic Experiments

Kinetic Experiments for the optimal AQ loading in the depolymerization of PMMA were conducted following the procedure established for BP (Section 2.2.1.1), with stock solutions prepared as mentioned above in Section 2.2.2.1. For NMR kinetic studies, the reaction was performed in a 20 mL Schlenk flask following the same general procedure.

Aliquots were periodically withdrawn under a nitrogen blanket at 1, 2, 4, and 8 h as shown in **Scheme S2**. Following each sampling event, the tubes were returned to the oil bath to continue the reaction.

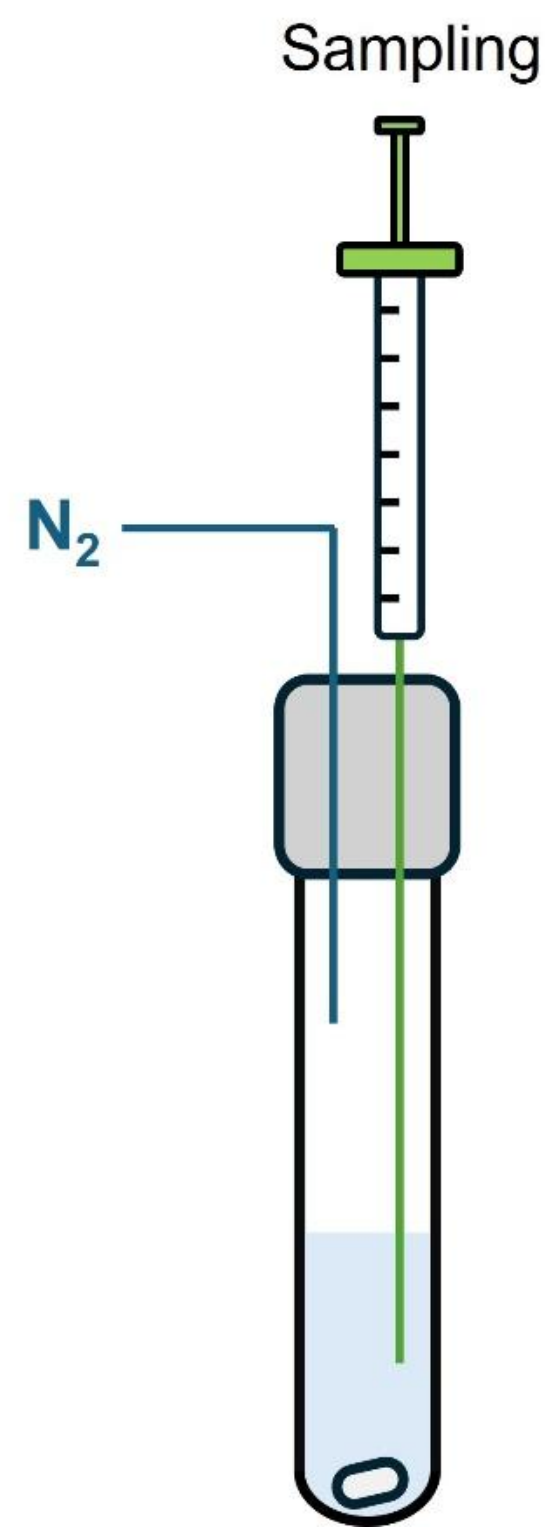


**Scheme S2** Kinetic Experiment Sampling Setup

### 2.2.3 Depolymerization of the Commercial PMMAs

The PMMA commodities were shredded prior to usage to facilitate dissolution.

The depolymerization of commercial PMMAs using AQ as the HAT agent was conducted following the procedure established for BP (section 2.2.1.1) with stock solutions prepared as follows:

20 mg of commercial PMMA (shredded) was dissolved in 10 mL of benzonitrile at 60 °C overnight to make the PMMA stock solution. In a separate vial 2.1 mg of AQ was dissolved in 5 mL of benzonitrile.

To prepare the depolymerization solution, 1 mL PMMA stock solution, 0.1 mL AQ stock solution, and 0.9 mL benzonitrile were mixed in a 10 mL glass tube to reach the initial concentrations ($[RU_0]$ = 10 mM, $RU_0$ : AQ = 1 : 0.01).

#### 2.2.3.1 Monomer Isolation

To a 50 mL Schlenk round-bottom flask, shredded pieces of transparent Plexiglas ($M_n$ = 380k, 1.5 g) were dissolved in purified benzonitrile (10 mL) overnight at 60 °C. Upon complete

dissolution of the polymer, a solution of anthraquinone (AQ, 31.2 mg, 0.01 equiv. relative to the repeating units [RU]) in purified benzonitrile (10 mL) was added. The resulting mixture was stirred for 10 min to ensure homogeneous distribution of the photocatalyst.

The flask was subsequently integrated into a distillation setup consisting of a distillation head equipped with a thermometer (to monitor the vapor temperature at the joint), a water-cooled condenser, and a receiving flask. The entire assembly was thoroughly deoxygenated via $N_2$ sparging for 30 min. To initiate the reaction, the reaction flask was immersed in an oil bath stationed within a photoreactor chamber. The area around the distillation joint was wrapped in aluminum foil to minimize heat loss. The reaction was allowed to proceed for 24 h to achieve simultaneous depolymerization and *in situ* distillation of the regenerated monomer.

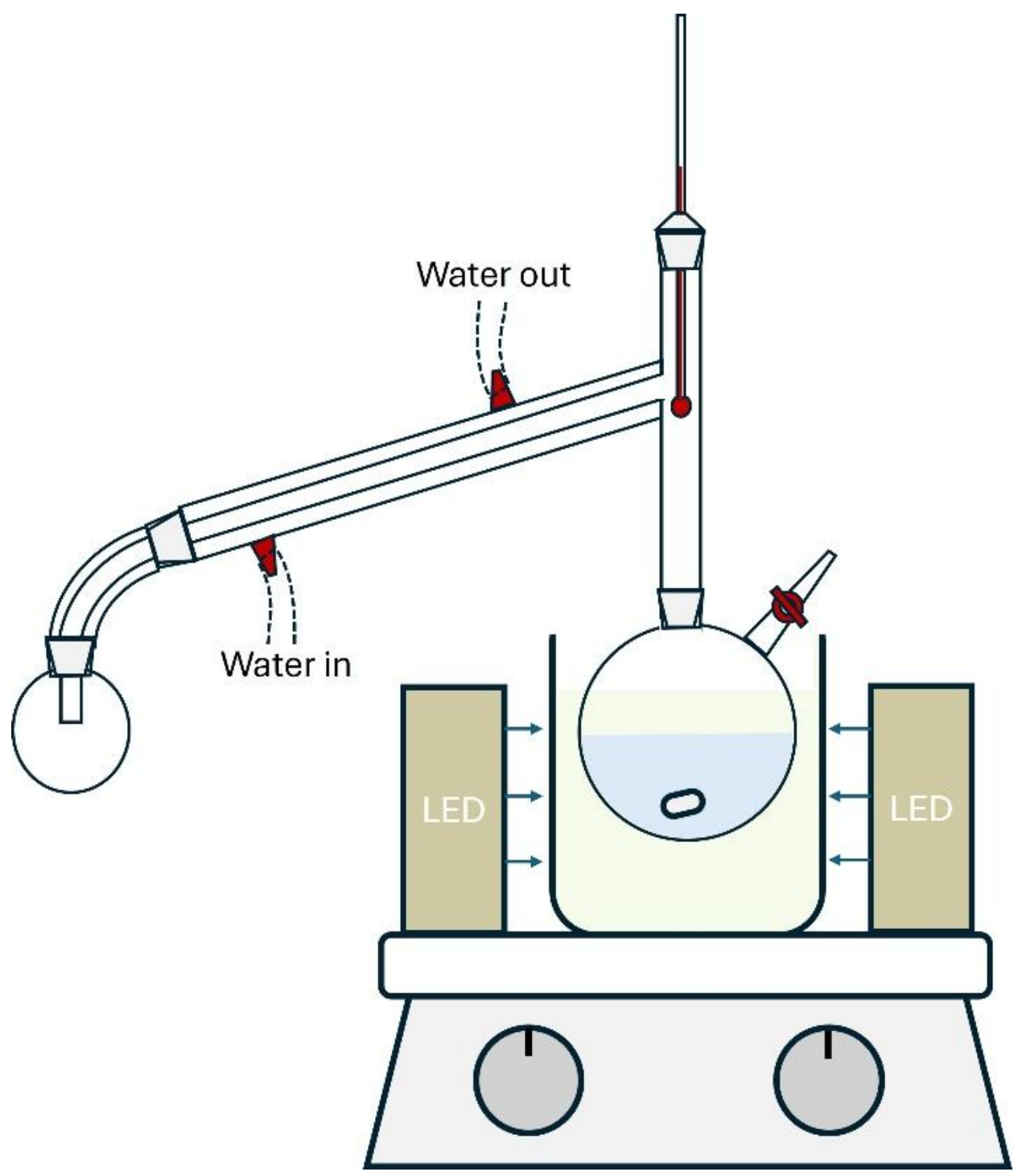


**Scheme S3** In situ depolymerization-distillation setup

### 2.2.4 Temporal Control of Depolymerization of PMMA

The temporal control of depolymerization of PMMA using AQ as the HAT agent was conducted following the procedure established for BP (section 2.2.1.1) with stock solutions prepared as follows:

20 mg of PMMA was dissolved in 10 mL of benzonitrile, and 2.1 mg of AQ was dissolved in 5 mL of benzonitrile. 2 mL PMMA stock solution, 0.2 mL AQ stock solution, and 1.8 mL benzonitrile were mixed in a 10 mL glass tube to reach the initial concentrations ([$RU_0$] = 10 mM, $RU_0$ : AQ = 1 : 0.01).

The reaction was subjected to three 'on/off' cycles. During the 'off' periods, both thermal and photo stimuli were removed; during the 'on' periods, both stimuli were restored. The reaction was ultimately quenched after 23 h by removing the vessel from the heat and light sources. Aliquots (0.3 mL) were collected under a nitrogen atmosphere at the end of each 'on' and 'off' interval for SEC analysis.

## 3 Additional Data

### 3.1 Characterization of PMMA Synthesized via FRP

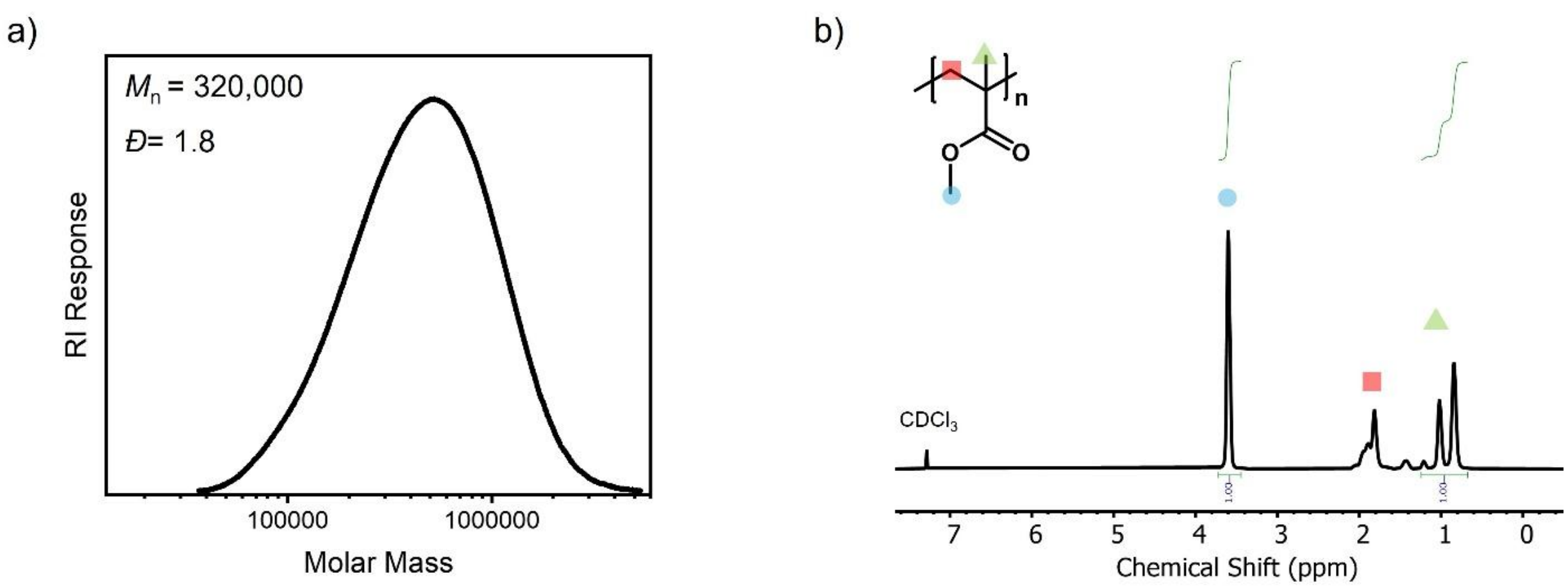


**Figure S1** Characterization of PMMA: a) SEC trace ($M_n$ = 320,000, $Đ$ = 1.8), b) $^1$H NMR spectrum after purification.

### 3.2 Solvent Selection for HAT Reaction

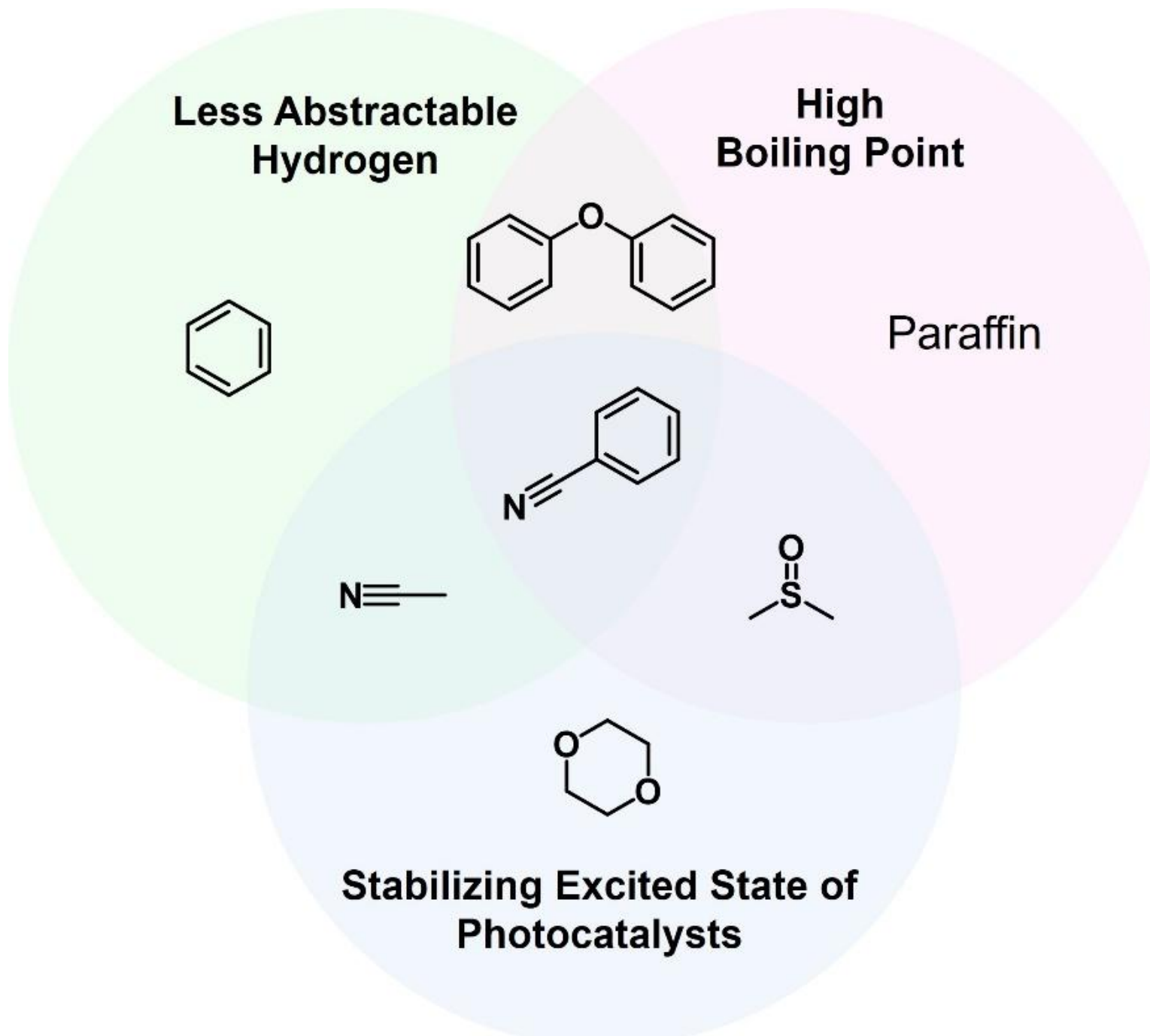


**Figure S2** Venn diagram for solvent selection

### 3.3 Control Experiments – Depolymerization without AQ

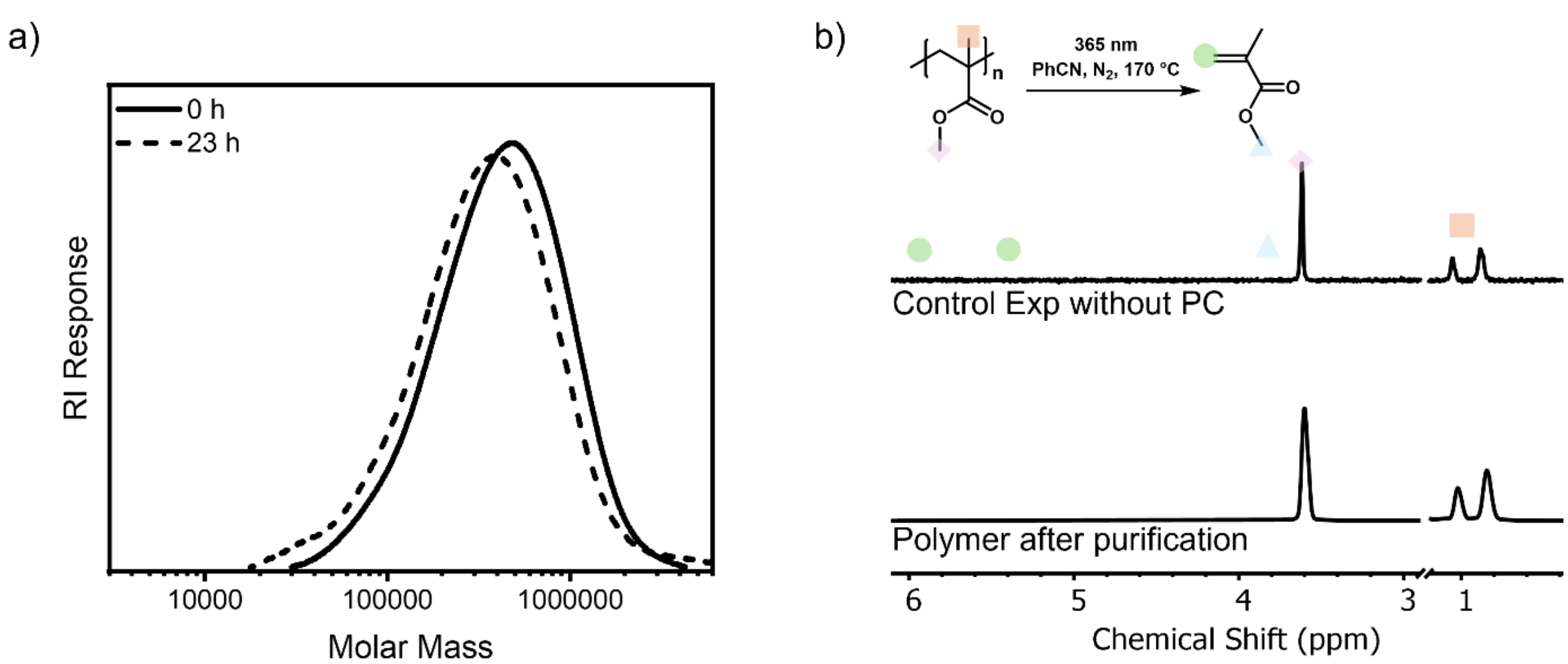


**Figure S3** Control experiment with purified benzonitrile. RUC of 10 mM PMMA in benzonitrile was reacted for 23 h at 170 °C with 365 nm light. a) SEC traces of $t_{0h}$ and $t_{23h}$. b) $^1$H NMR showed a negligible amount of monomer recovery.

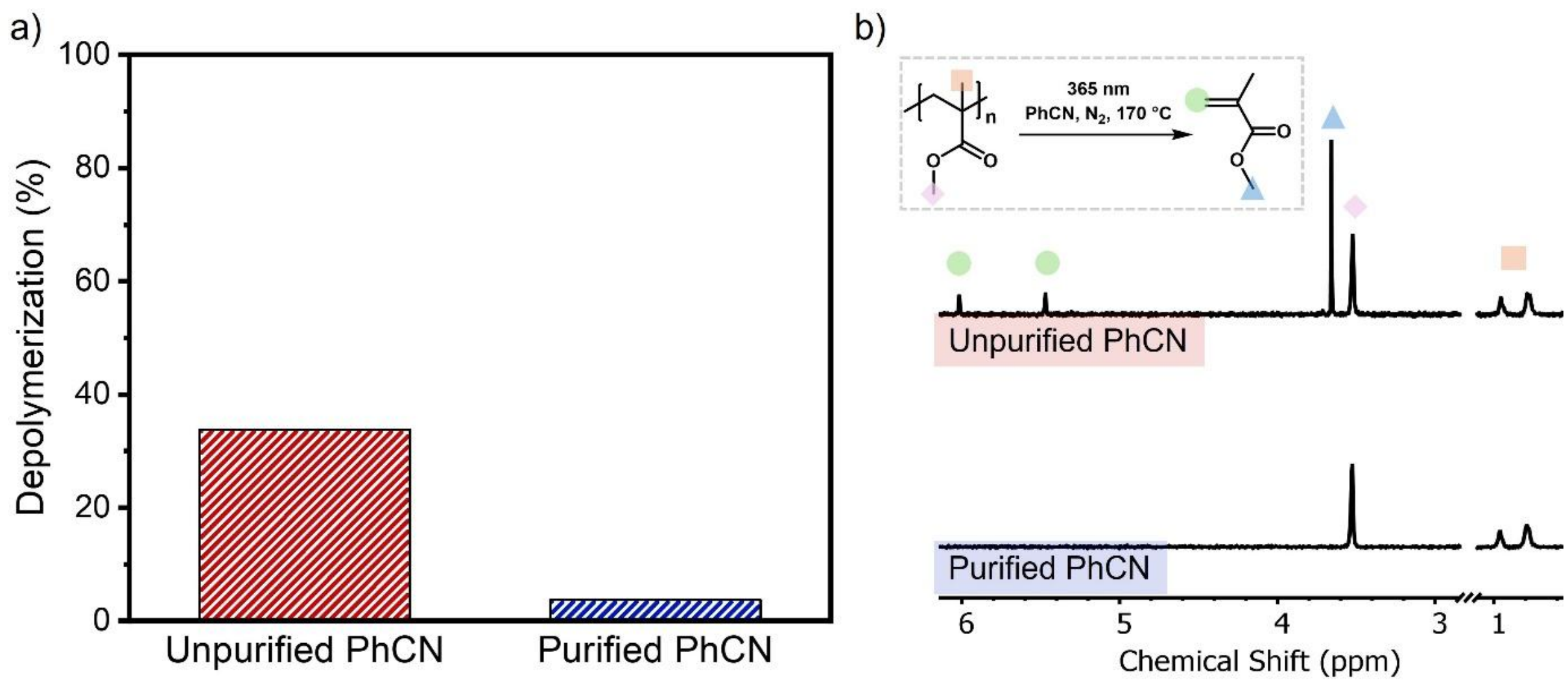


**Figure S4** Control experiment with unpurified benzonitrile (unpurified PhCN) and purified benzonitrile (purified PhCN). RUC of 10 mM PMMA in benzonitrile was reacted for 23 h at 170 °C with 365 nm light. a) Depolymerization conversion measured by SEC. b) $^{1}H$ NMR showed approximately 30% monomer regeneration with unpurified PhCN, while nearly no monomer was recovered with purified PhCN.

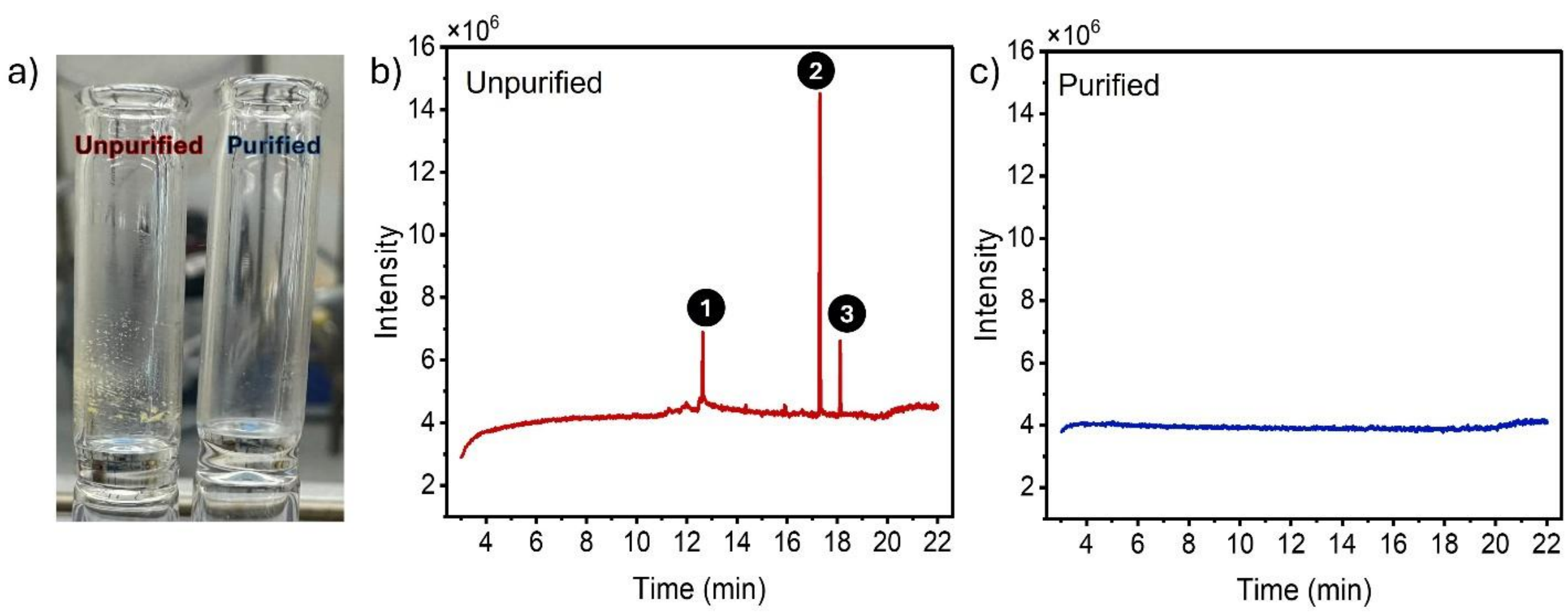


**Figure S5** Evidence for removal of impurities in benzonitrile. a) Photographs of residues remaining after air-drying 100 mL of benzonitrile without purification (left) and after purification with basic alumina (right). b) Gas chromatogram of unpurified benzonitrile, showing three main identified compounds. c) Gas chromatogram of purified benzonitrile.

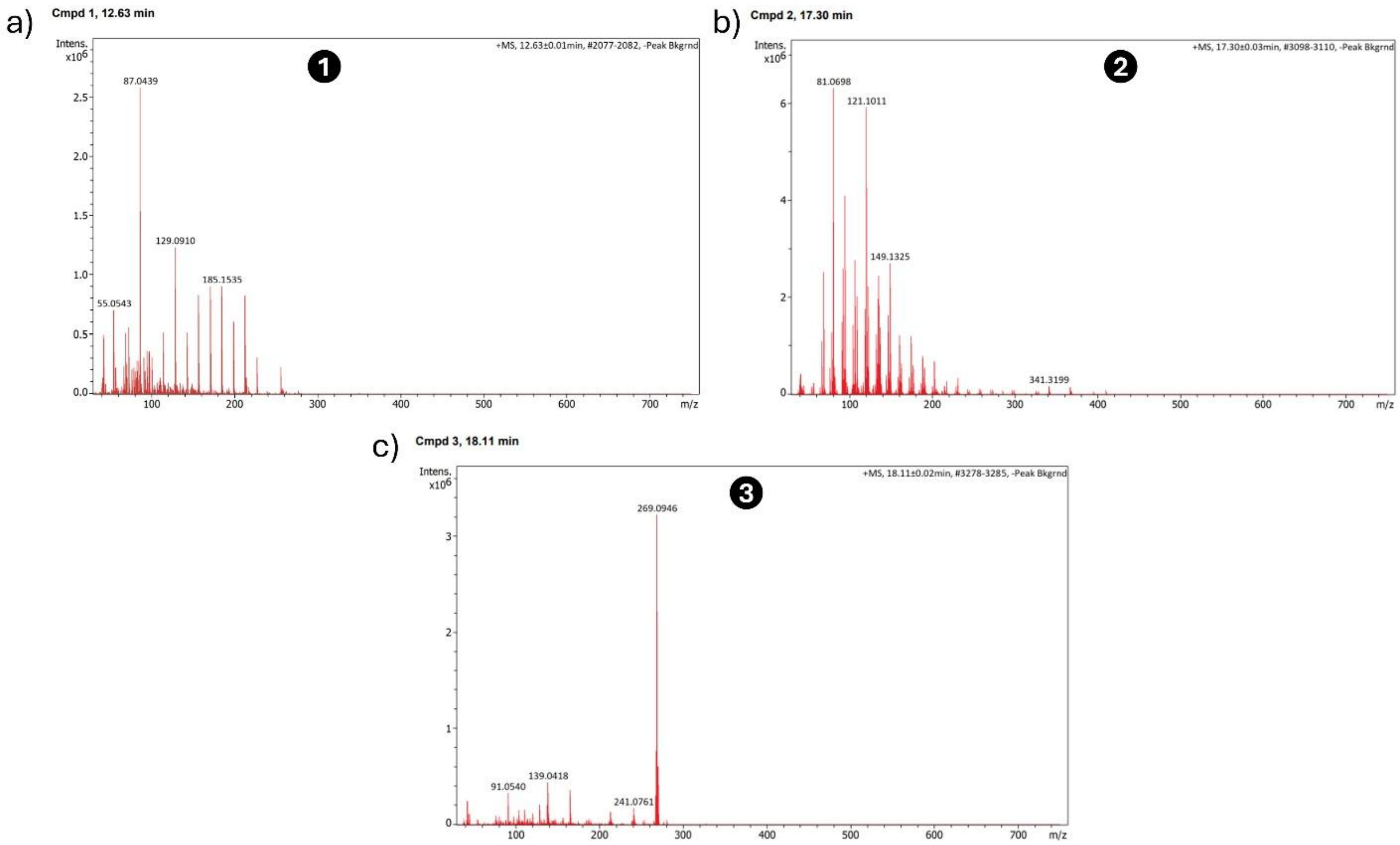


**Figure S6** Identification of impurities in benzonitrile. a) Mass spectrum corresponding to the first peak in the unpurified benzonitrile gas chromatogram. b) Mass spectrum corresponding to the second peak in the unpurified benzonitrile gas chromatogram. c) Mass spectrum corresponds to the third peak in the unpurified benzonitrile gas chromatogram.

### 3.4 Photochemical Properties of Aromatic Ketones

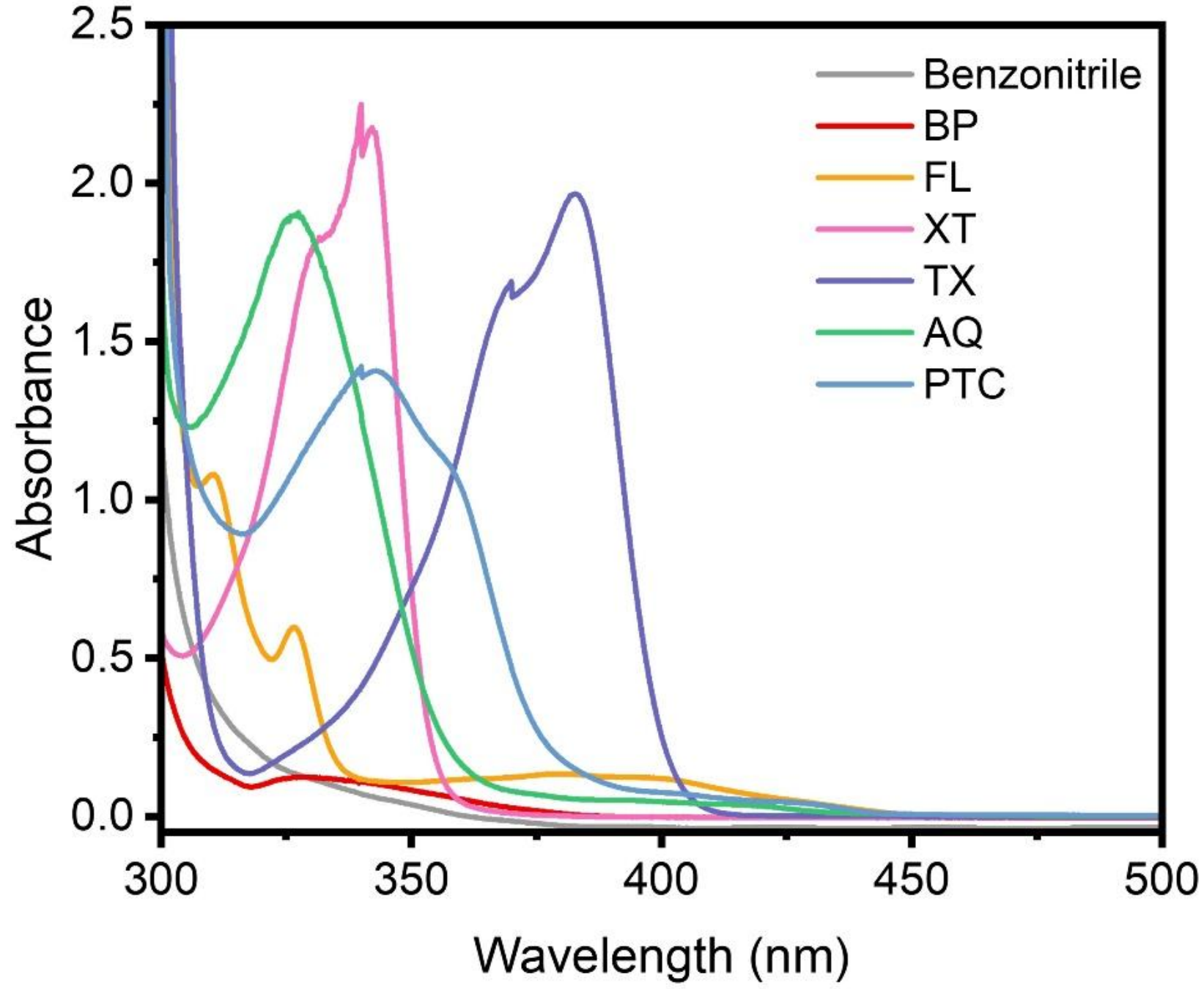


**Figure S7** UV-vis spectra of the aromatic ketones and benzonitrile. Baseline for aromatic ketones was benzonitrile, and baseline for benzonitrile was air. Solution concentrations were 0.3 mM for BP, FL, TX, AQ, and PTC, and 0.17 mM for XT.

**Table S2** Maximum absorbance, molar absorptivity, and reported triplet energy of aromatic ketones.

| | BP | FL | XT | TX | AQ | PTC |
|---|---|---|---|---|---|---|
| Max Absorbance (nm) | ~330 | ~320 | ~350 | ~ 390 | ~330 | ~350 |
| Molar Absorptivity at 365 nm[a] ($M^{-1}cm^{-1}$) | 133.6 | 718.9 | 67.2 | 5013 | 350.5 | 2556 |
| Triplet Energy (kcal/mol) | 68.5[1] | 67.6[1] | 74.2[1] | 65.5[1] | 62.4[1] | -- |

[a] Calculated according to the Beer–Lambert law (A = εlc), where A is the absorbance at 365 nm in **Figure S7**, l is the path length, c is the molar concentration, and ε is the molar absorptivity.

### 3.5 Photocatalyst Screening for Depolymerization of PMMA

**Table S3** Depolymerization Conversions and $M_n$ (at $t_{0h}$ and $t_{8h}$) of Photocatalyst Screening§

| PC | Depolymerization (%)* | $M_n$, $t_{0h}$ | $M_n$, $t_{8h}$ |
|---|---|---|---|
| No PC (PhCN) | 4 | 322,000 | 296,500 |
| BP | 4 | 322,000 | 265,400 |
| FL | 5 | 322,000 | 214,300 |
| XT | 10 | 322,000 | 241,800 |
| TX | 43 | 322,000 | 127,900 |
| AQ | 83 | 322,000 | 33,200 |
| PTC | 63 | 322,000 | 77,700 |

§ RUC of 10 mM PMMA and 0.025 eq aromatic ketones ($RU_0$: Aromatic ketone = 1: 0.025) in benzonitrile were reacted for 8 h at 170 °C with 365 nm light.

* Depolymerization conversions were calculated via SEC analysis, using the following formula: Conversion = $(1-(Area_t/Area_{t0}))*100$, where $t_{0h}$ and $t_{xh}$ denote samples taken at time 0 h and x h.

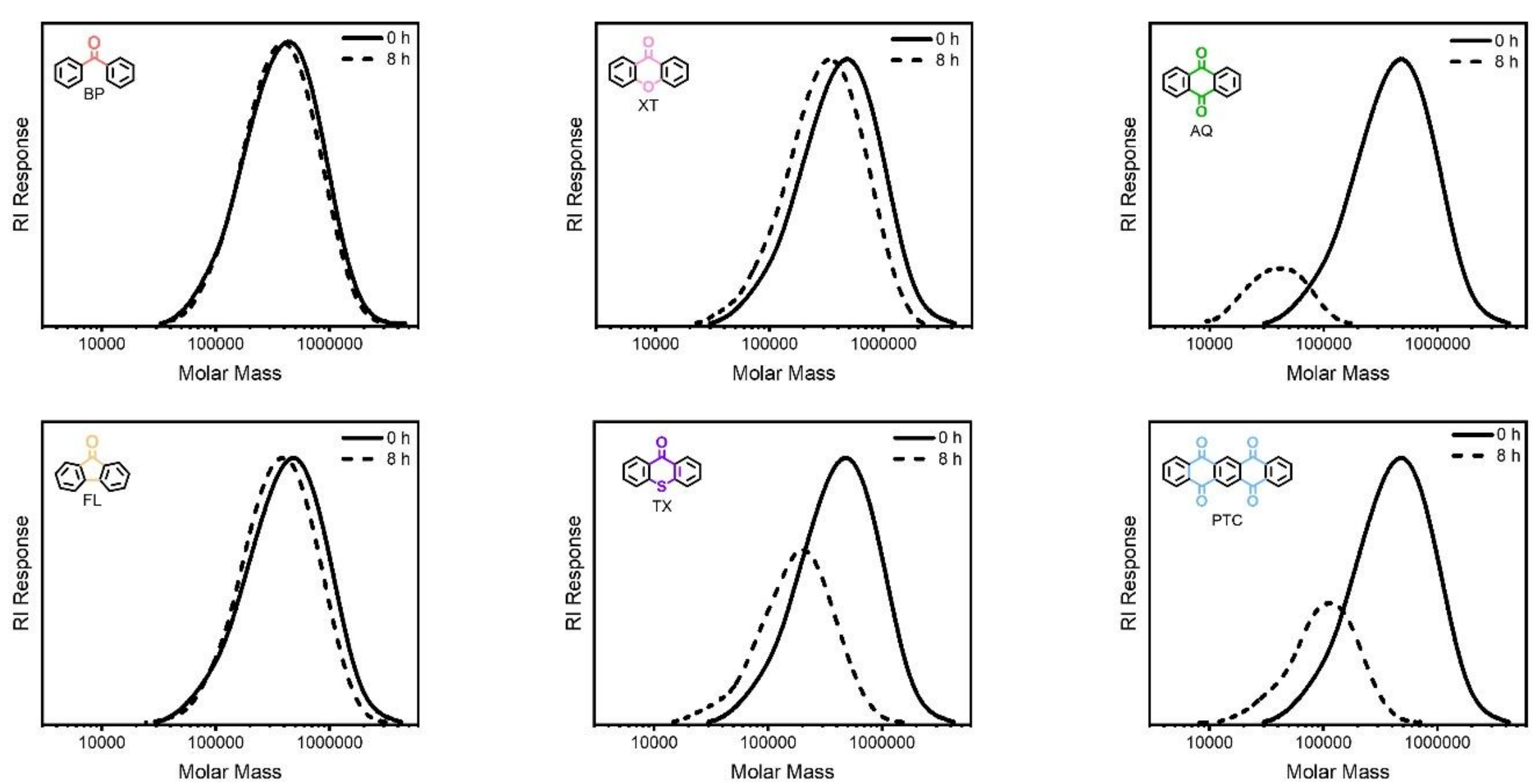


**Figure S8** Evolution of molecular weight distributions during the aromatic ketone-mediated depolymerization of PMMA, as monitored by SEC. RUC of 10 mM PMMA and 0.025 eq aromatic ketones in benzonitrile were reacted for 8 h at 170 °C with 365 nm light. Significant concurrent chain scission and depolymerization happened in the reaction with TX, AQ, and PTC.

### 3.6 Optimization of AQ Loadings

**Table S4** Evolution of depolymerization conversion and $M_n$ compared to the initial polymer for the depolymerization of PMMA using X (where X = 0.005-0.1) eq AQ§

| PC loading (eq) | Depolymerization (%)* | $M_n$, $t_{0h}$ | $M_n$, $t_{8h}$ |
|---|---|---|---|
| 0.005 | 31 ± 0 | 313,900 | 51,600 |
| 0.0075 | 73 ± 0 | 313,900 | 41,500 |
| 0.01 | 94 ± 2 | 313,900 | 18,300 |
| 0.025 | 84 ± 1 | 313,900 | 16,600 |
| 0.05 | 69 ± 6 | 313,900 | 13,200 |
| 0.1 | 48 ± 3 | 313,900 | 10,800 |

§ RUC of 10 mM PMMA and X eq aromatic ketones ($RU_0$: AQ = 1: X, X = 0.005-0.1) in benzonitrile were reacted for 8 h at 170 °C with 365 nm light.

* Depolymerization conversions were calculated via SEC analysis, using the following formula: Conversion = (1-($Area_t$/$Area_{t0}$))*100, where $t_{0h}$ and $t_{xh}$ denote samples taken at time 0 h and x h.

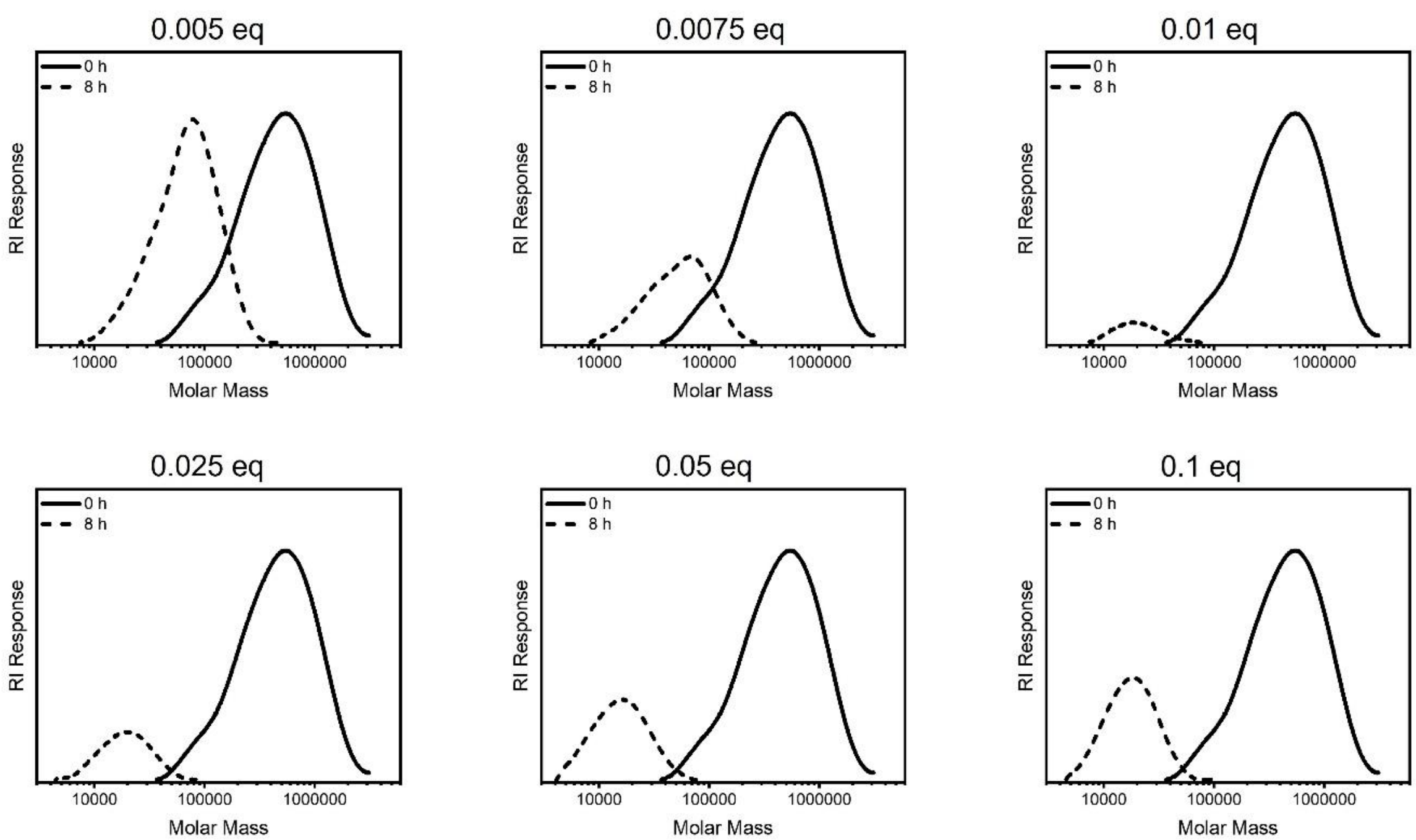


**Figure S9** SEC curves of depolymerization with varying amounts of AQ. RUC of 10 mM PMMA and 0.005-0.1 eq aromatic ketones in benzonitrile were reacted for 8 h at 170 °C with 365 nm light.

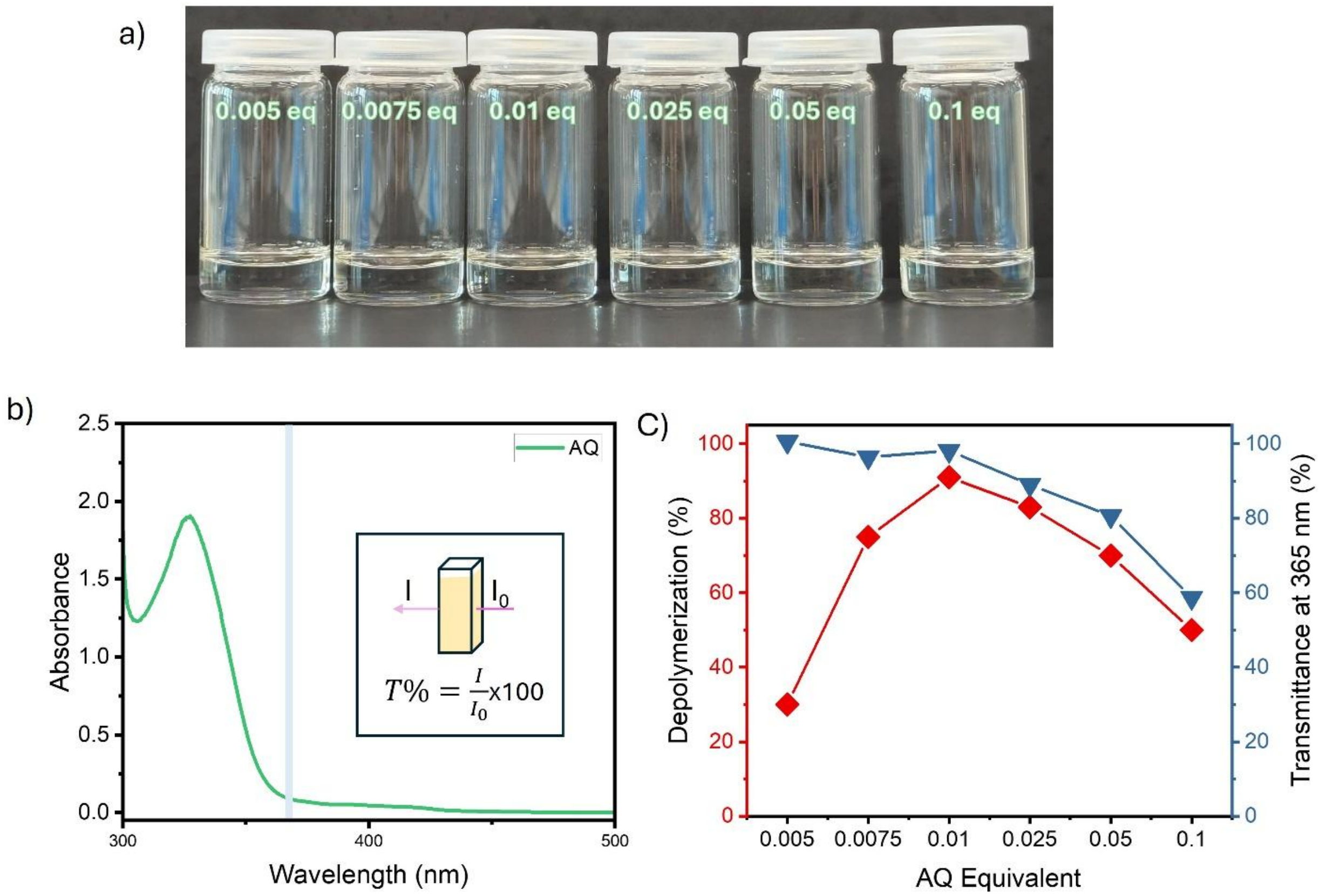


**Figure S10** Solubility and transmittance profiles of AQ in benzonitrile. (a) Visual appearance of the reaction mixtures showing full catalyst solubility across all tested equivalents. (b) UV-Vis absorption spectrum of AQ in benzonitrile; the target excitation wavelength (365 nm) is highlighted in blue. (c) Plot illustrating the relationship between depolymerization efficiency and solution transmittance at 365 nm as a function of catalyst loading.

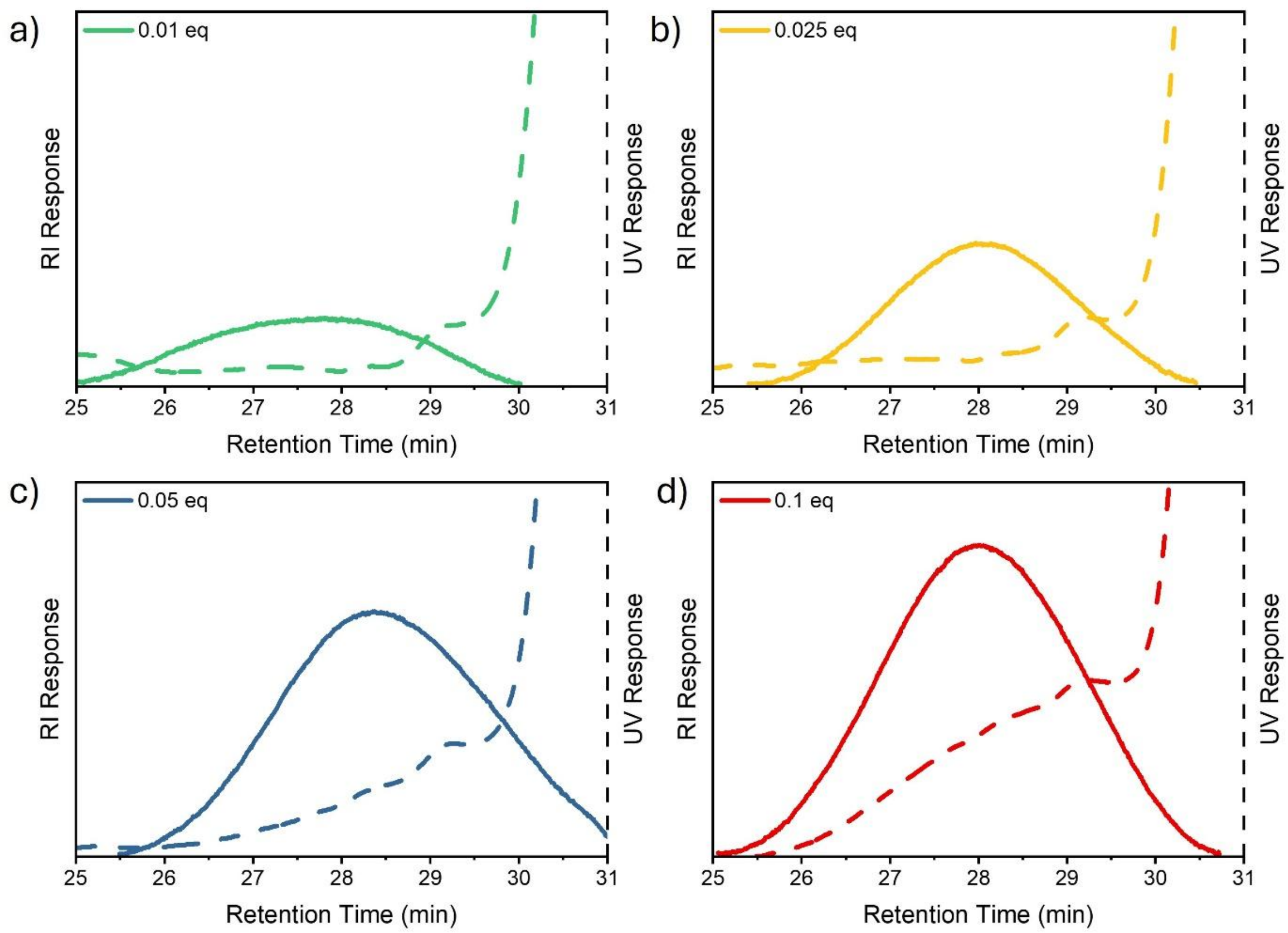


**Figure S11** SEC traces of the polymers after 24 h of reaction across catalyst loadings from 0.01 to 0.1 equiv of AQ. The increase in the UV signal intensity relative to the refractive index (RI) traces indicates the incorporation of UV-active molecules into the polymer backbone.

### 3.7 Kinetic Profile of the Depolymerization with Optimized AQ Loading

**Table S5** Depolymerization conversions and molecular weight evolution for the depolymerization of PMMA using 0.01 eq AQ.[§]

| Time (h) | Depolymerization conversion by SEC (%)[*] | $M_n$ | Depolymerization conversion by NMR (%)[a] |
|---|---|---|---|
| 0 | 0 | 313,900 | 0 |
| 1 | 32 | 131,800 | 27 |
| 2 | 46 | 105,800 | 45 |

| | | | |
|---|---|---|---|
| 4 | 67 | 74,400 | 66 |
| 8 | 98 | 25,300 | 96 |

§ RUC of 10 mM PMMA and 0.01 eq aromatic ketones ($RU_0$ : Aromatic ketone = 1: 0.01) in benzonitrile were reacted for 23 h at 170 °C with 365 nm light.

* calculated via SEC analysis, using the following formula: Conversion = (1-($Area_t$/$Area_{t0}$))*100, where $t_{0h}$ and $t_{xh}$ denote samples taken at time 0 h and x h.

[a] calculated via $^{1}$H NMR analysis.

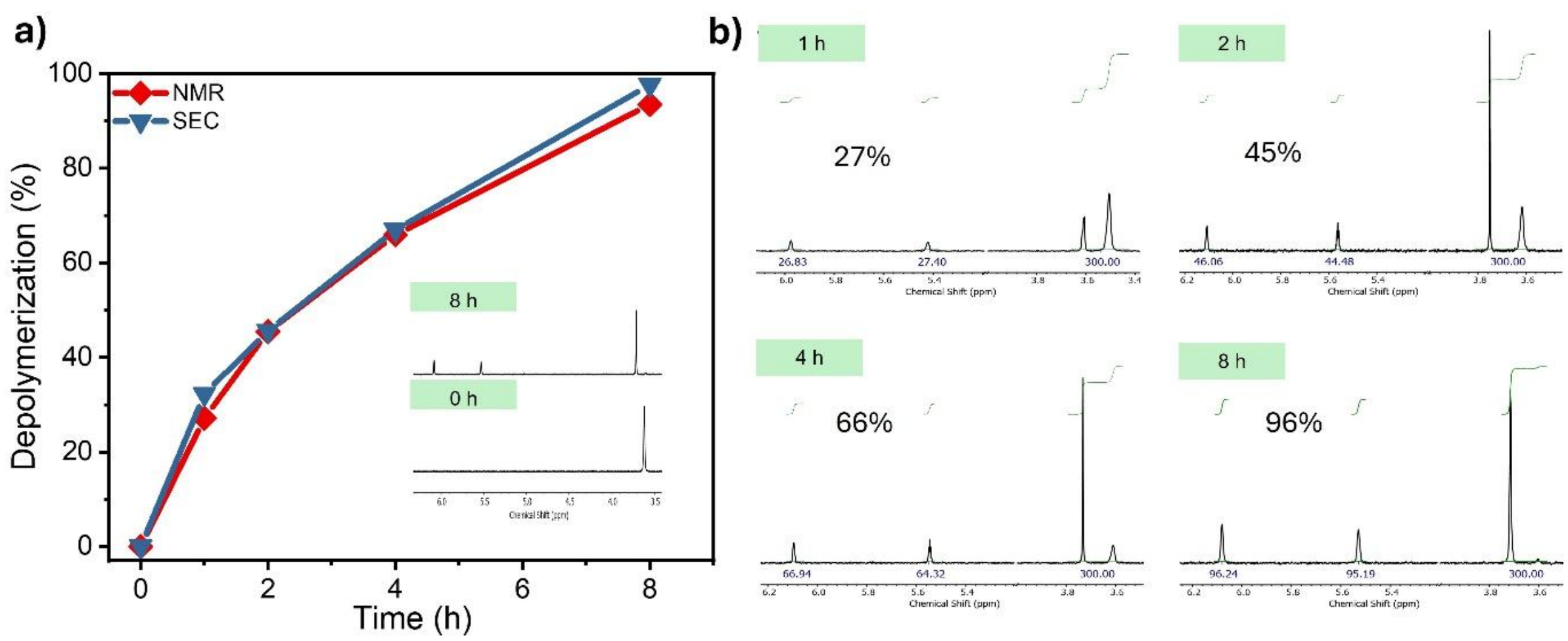


**Figure S12** Depolymerization profile of RUC PMMA. Conditions: 10 mM RUC PMMA in benzonitrile at 170 °C, 0.01 eq. AQ, under 365 nm irradiation. (a) Kinetic profile comparing monomer conversions determined via SEC and $^1$H NMR spectroscopy (inset: $^1$H NMR spectra of the reaction mixture before (0 h) and after (8 h) depolymerization). (b) $^1$H NMR spectra showing the evolution of the polymer methoxy peaks (3.6–3.8 ppm, integrated to 300) and the emerging vinyl peaks of the regenerated MMA monomer (5.5 and 6.1 ppm) at 1, 2, 4, and 8 h, corresponding to 27%, 45%, 67%, and 96% conversion, respectively.

## 3.8 Proposed Mechanism of Oxygen-Centered HAT Agent Triggered Mid-Chain Depolymerization of PMMA

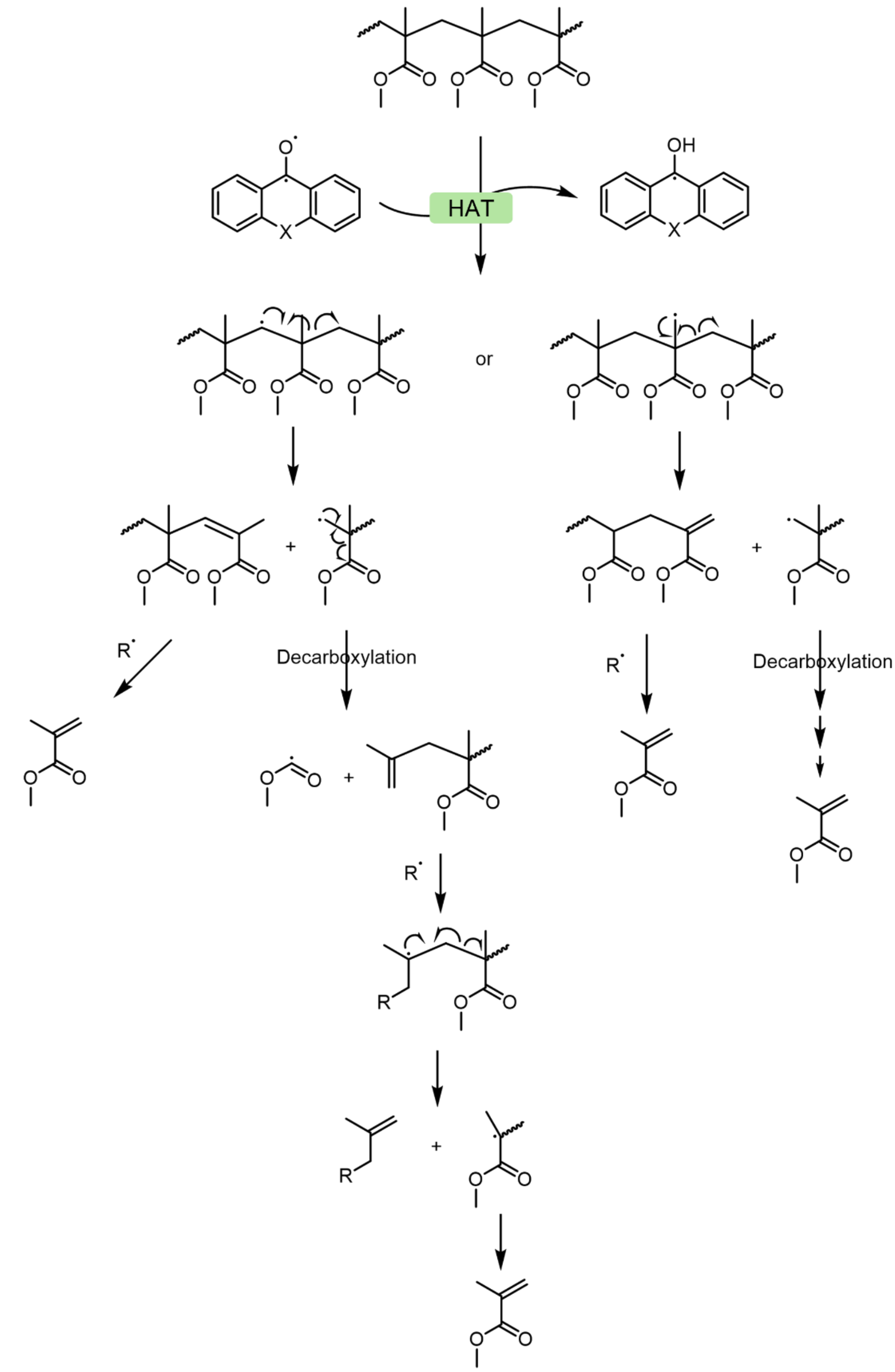


**Figure S13** Proposed mechanism for mid-chain scission and depolymerization via triplet biradical with oxygen-centered reactivity derived from aromatic ketones.

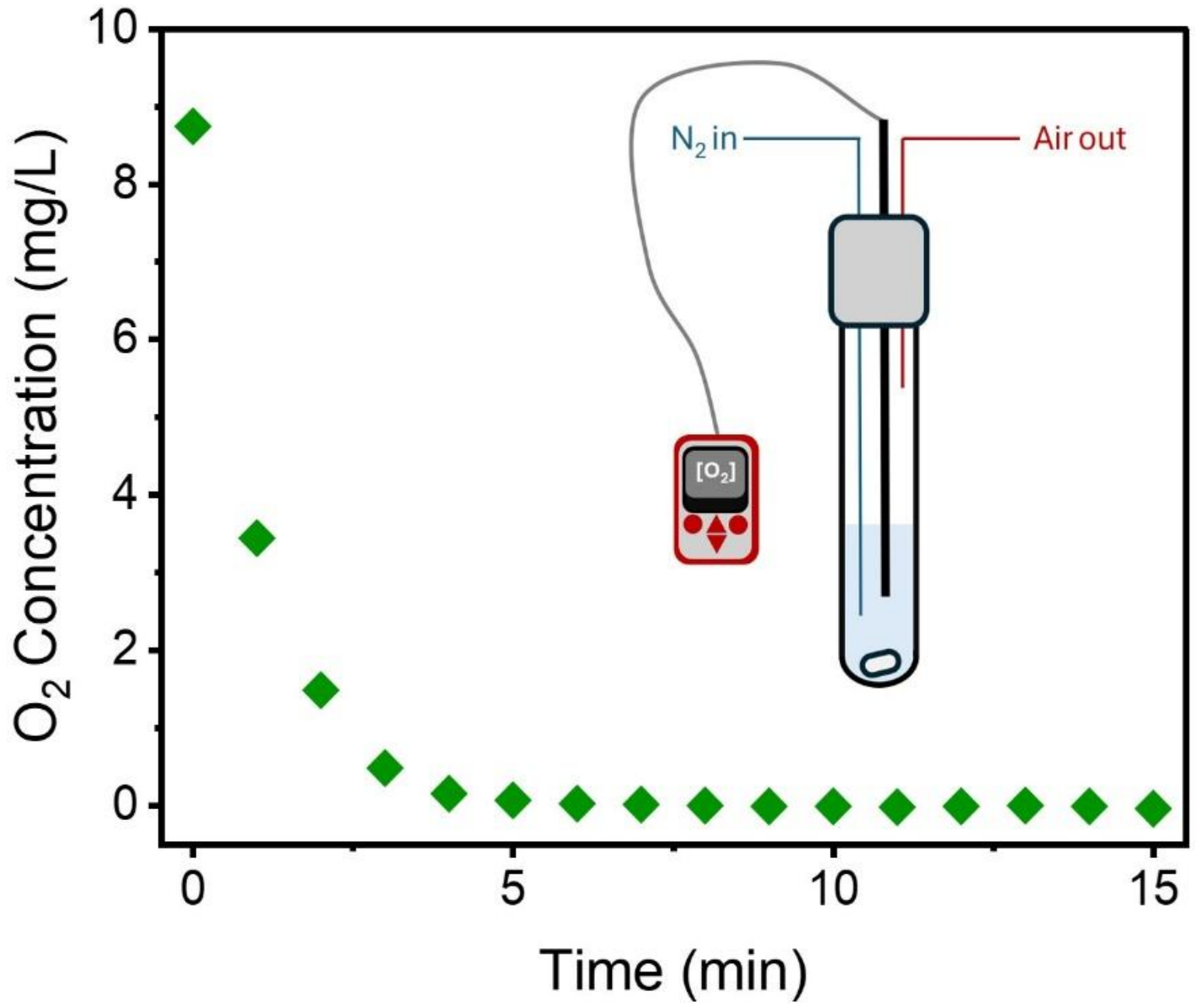


**Figure S14** Dissolved oxygen concentration as a function of $N_2$ purging time during the degassing phase prior to reaction (15min).

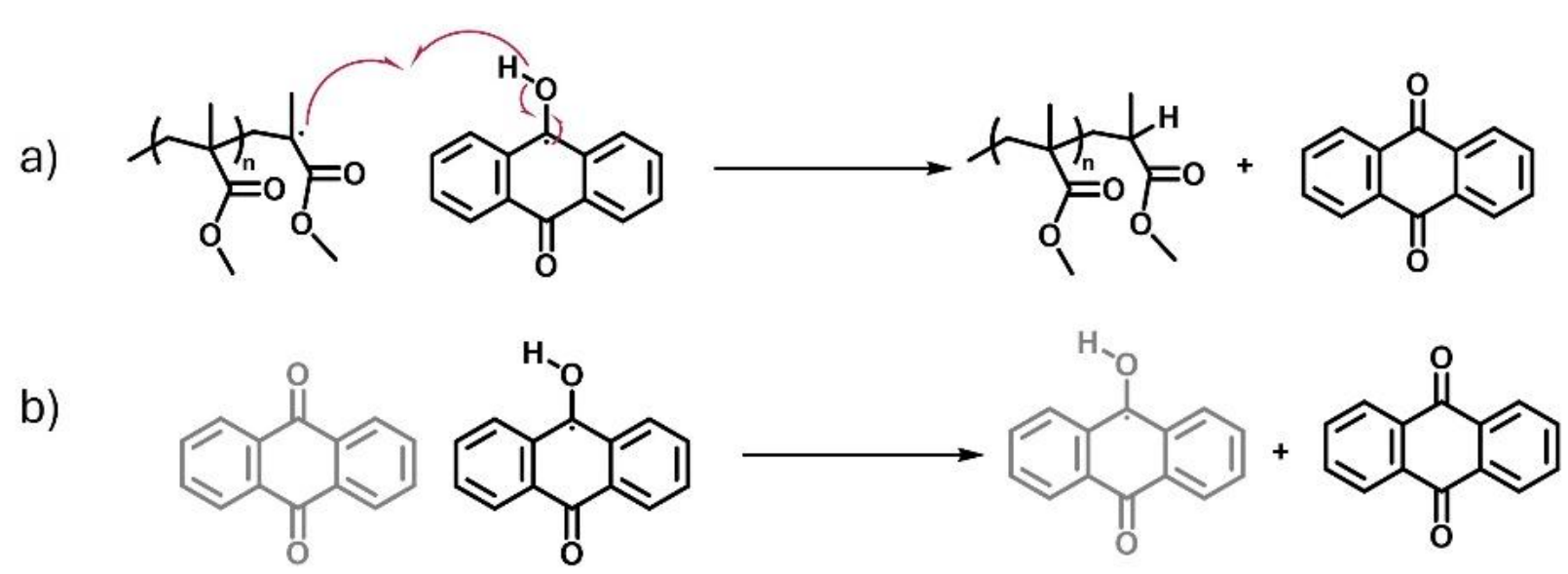


**Figure S15**: Proposed AQ regeneration pathways. a) Reverse HAT process with a polymeric radical, b) Disproportionation of the ·PC-H with another PC molecule

## 3.9 Radical Electrophilicity and Bond Dissociation Energies

**Table S6** Electrophilicity of chlorine radical and triplet biradical with oxygen-centered reactivity.[2]

| Radical | Electrophilicity (eV) |
|---|---|
| Cl· | 3.8 |

| | |
|---|---|
| 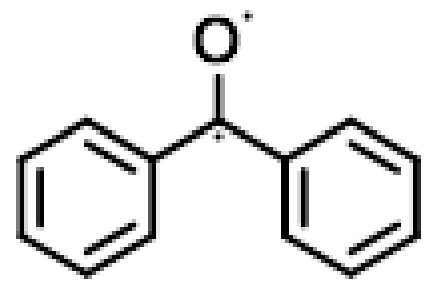 | 4.13† |

†Electrophilicity of oxygen-centered radical in the triplet form of aromatic ketones.[2]

**Table S7** Comparison of C–H bond dissociation energies (BDE) across diverse chemical environments. [3]

| C-H Bond | Bond Dissociation Energy (kcal/mol) |
|---|---|
| $(C_6H_5)$-H [b] | 113 |
| **H**-$CH_2CH_3$ [a] | 101 |
| **H**-$CH(CH_3)_2$ [a] | 98 |
| H-Cl [c] | 103 |
| H-OR [abc] | 104 |

[a] BDE of H-OR is larger than BDE of H-$CH_2CH_3$ and H-$CH(CH_3)_2$, showing the potential of oxygen-centered radicals abstracting hydrogens from methyl and methylene units.

[b] BDE of $(C_6H_5)$-H is much larger than BDE of H-$CH_2CH_3$, H-$CH(CH_3)_2$, and H-OR, implying that the hydrogens on aryl rings may be less abstractable than the ones on methyl and methylene units to oxygen-centered radicals.

[c] BDE of H-OR is slightly higher than BDE of H-Cl, indicating that oxygen-centered radicals might possess the capability of conducting HAT reaction similar to chlorine-centered radicals.

## 3.10 Depolymerization of Commercial PMMAs

**Table S8** Depolymerization Conversion and $M_n$ of Depolymerization of Commercial PMMAs

| Commercial PMMAs | Depolymerization (%)* | $M_n$, $t_{0h}$ | $M_n$, $t_{8h}$ |
|---|---|---|---|
| r-PG† | 96 | 165,800 | 21,200 |
| b-PG† | 97 | 205,800 | 26,700 |
| y-PG† | 91 | 92,800 | 24,600 |
| g-PG† | 94 | 131,200 | 20,300 |
| AT‡ | 91 | 58,500 | 28,700 |
| LGP‡ | 92 | 57,500 | 15,600 |

†RUC of 10 mM commercial PMMA and 0.01 eq aromatic ketones ($RU_0$ : Aromatic ketone = 1: 0.01) in benzonitrile were reacted for 72 h at 170 °C with 365 nm light.

‡ RUC of 10 mM commercial PMMA and 0.01 eq aromatic ketones ($RU_0$ : Aromatic ketone = 1: 0.01) in benzonitrile were reacted for 120 h at 170 °C with 365 nm light.

* Depolymerization conversions were calculated via SEC analysis, using the following formula: Conversion = (1-($Area_t/Area_{t0}$))*100, where $t_{0h}$ and $t_{xh}$ denote samples taken at time 0 h and x h.

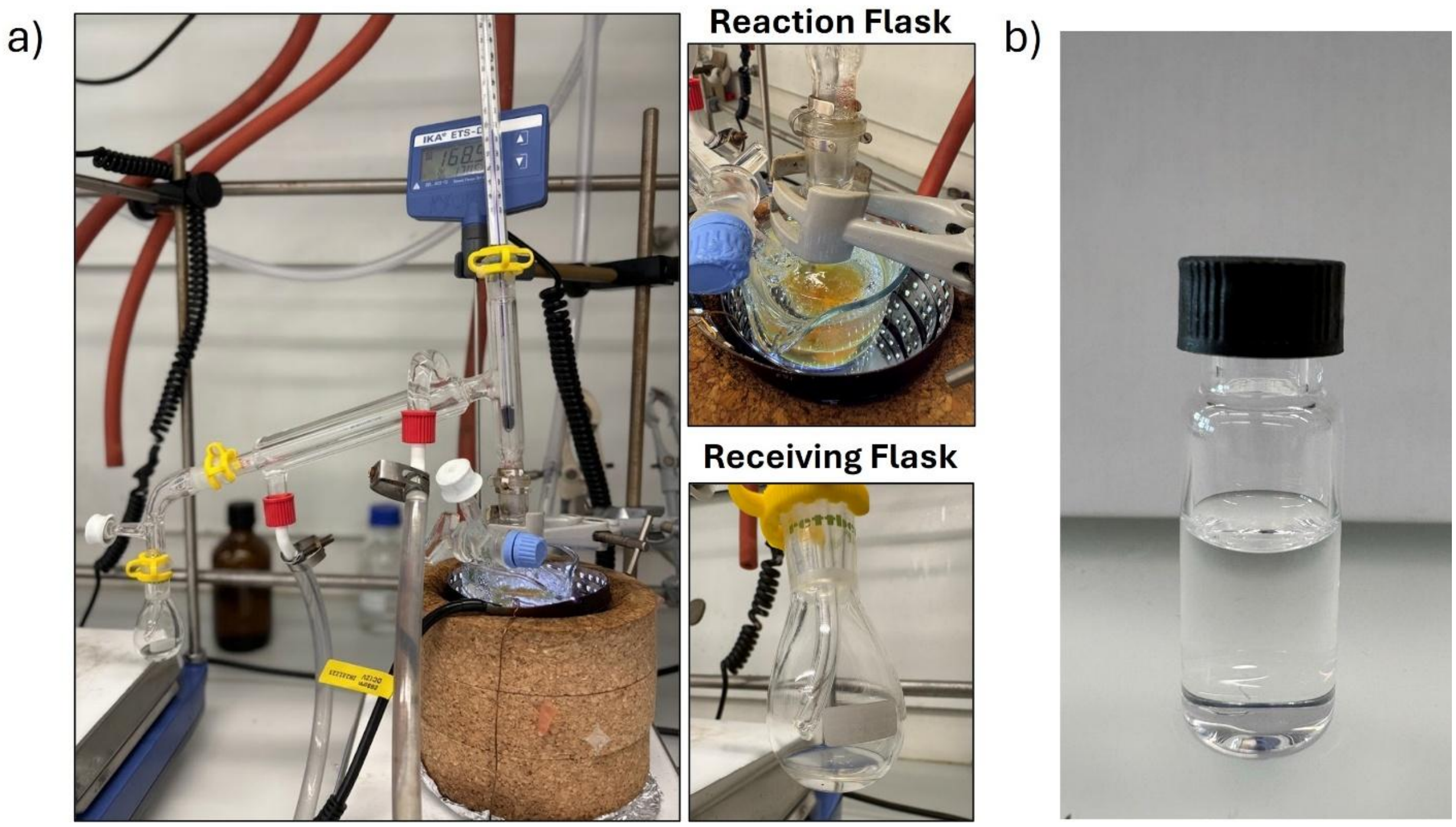


**Figure S16** Isolation of monomer. (a) *In situ* distillation apparatus used for the recovery of monomer during depolymerization under 365 nm light at 170 °C (reaction conditions: 750 mM [RUC], 0.01 eq. AQ). (b) Photograph of the collected liquid monomer (1.08 g) in a vial.

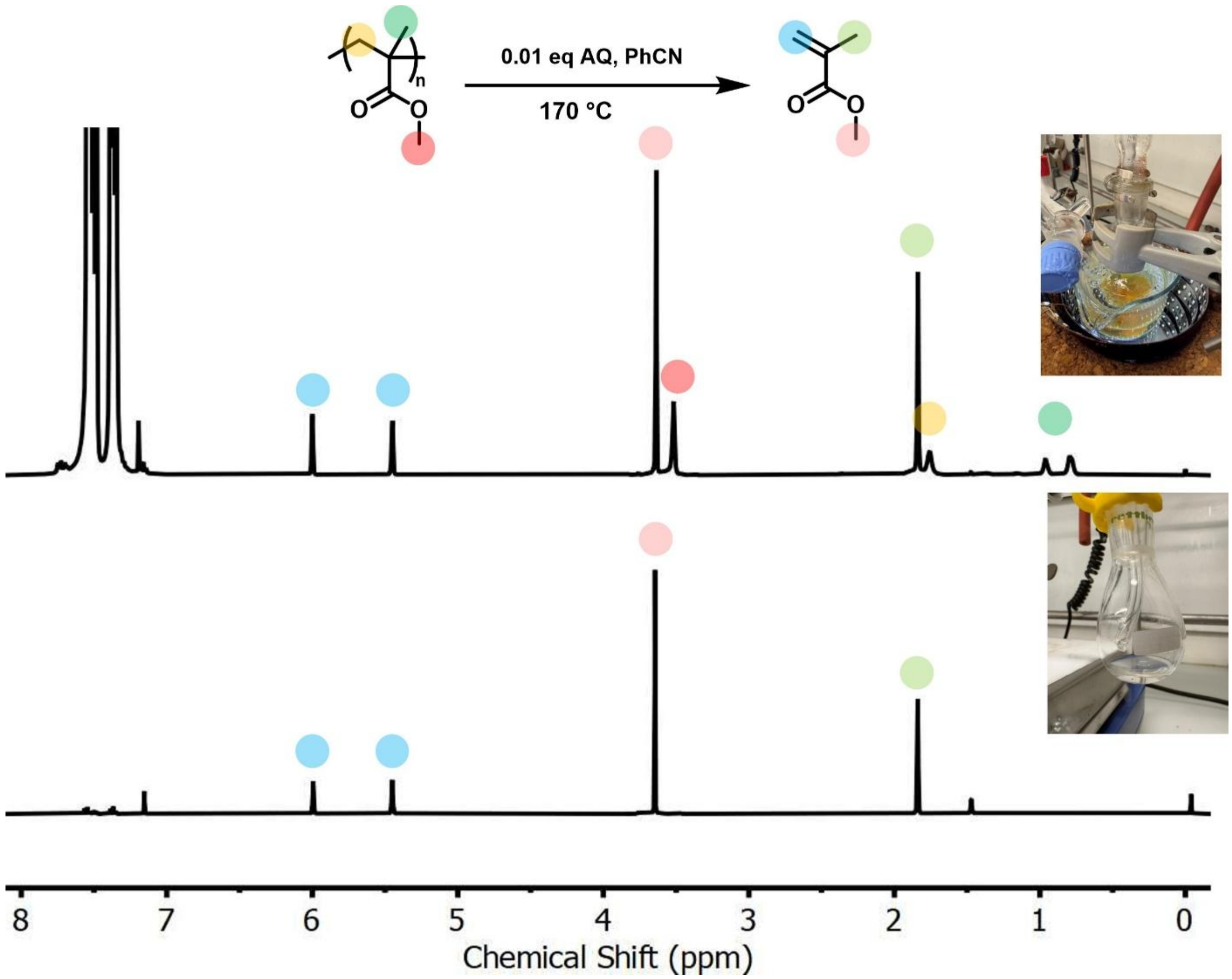


**Figure S17** Characterization of the isolated monomer**.** Top: $^{1}$H NMR spectrum of the reaction mixture (taken under $N_2$) at 8 h after initiating the reaction. Bottom: $^{1}$H NMR spectrum of the final collected liquid in the receiving flask, confirming the isolation of MMA monomer in ~95% purity; the remaining 5% is attributed to co-distilled PhCN (signals at 7.2–7.7 ppm).

## References


[1] W. G. Herkstroeter, A. A. Lamola, G. S. Hammond, "Mechanisms of photochemical reactions in solution. XXVIII. 1 Values of triplet excitation energies of selected sensitizers," *Journal of the American Chemical Society* **86**, (1964): 4537–4540.

[2] J. J. Garwood, A. D. Chen, D. A. Nagib, "Radical polarity," *Journal of the American Chemical Society* **146**, (2024): 28034–28059.

[3] Y.-R. Luo, *Comprehensive handbook of chemical bond energies*, CRC press, Boca Raton, (2007), pp. 19-147.
